# Global, regional, and national burden of acute myeloid leukemia attributable to tobacco from 1990 to 2021, with future forecasts to 2050: A secondary dataset analysis of the Global Burden of Disease 2021 study

**DOI:** 10.18332/tid/218783

**Published:** 2026-07-20

**Authors:** Jianhua Zhang, Linghua Zhao, Yao Chao, Zhijuan Zhang, Jianli Guo

**Affiliations:** 1 Department of Hematology, The Second Hospital of Shanxi Medical University, Taiyuan, China; 2 Department of Infection Control, Shanxi Cancer Hospital, Taiyuan, China

**Keywords:** acute myeloid leukemia, age-period-cohort, tobacco, Bayesian age-period-cohort model

## Abstract

**INTRODUCTION:**

Acute myeloid leukemia (AML) is a major public health problem. However, the epidemiological pattern of the burden of AML attributable to tobacco globally remains unclear. This study aimed to characterize the global, regional, and national burden of AML attributable to tobacco from 1990 to 2021 and to forecast it to 2050.

**METHODS:**

Data were sourced from the Global Burden of Disease (GBD) study 2021, and secondary dataset analysis was used in the present research. Age-standardized rates of tobacco-attributable AML deaths and disability-adjusted life years (DALYs) were analyzed, with estimated annual percentage change (EAPC) calculated. Age-period-cohort (APC) analysis was performed to quantify the age, period, and birth cohort effects on AML attributable to tobacco. A Bayesian APC model was subsequently us ed to project the disease burden of attributable to tobacco between 2022 and 2050.

**RESULTS:**

From 1990 to 2021, the burden of AML attributable to tobacco globally presented downward trends. The age-standardized death rate (ASDR) decreased from 0.214 [95% uncertainty interval (UI): 0.08-0.35] per 100000 people in 1990 to 0.176 (95% UI: 0.062–0.307) per 100000 people in 2021. The high sociodemographic index (SDI) regions had the highest ASDR and age-standardized DALYs rate for AML attributable to tobacco in 2021. The greatest burden of AML attributable to tobacco was the age group of 70–74 years among the risk factors of AML; tobacco shared at 11.5% of global deaths. As for the age effect, death due to AML attributable to tobacco increased with age, rising slowly before 40 years of age and accelerating sharply thereafter. The ASDR for males decreased from 0.3 per 100000 people in 2021 to 0.24 per 100000 people in 2022.

**CONCLUSIONS:**

Although the burden of AML attributable to tobacco had been on a downward trend from 1990 to 2021, it is particularly prominent in high SDI regions, high-income regions, and the elderly population.

## INTRODUCTION

Acute myeloid leukemia (AML) is a type of hematological malignancy in which myeloid hematopoietic stem/progenitor cell differentiation is impaired, and leukemic cells proliferate in a malignant clonal fashion^1^. AML is the most common acute leukemia in adults, and in 2021, there were 144645 new cases of AML globally with an age-standardized incidence rate of 1.73 cases per 100000 people and 130189 deaths with an age-standardized death rate (ASDR) of 1.57 cases per 100000 people^2^. In recent years, with the continuous advancement of chemotherapy, supportive care, hematopoietic stem cell transplantation, and other aspects, as well as the application of new targeted drugs, the prognosis of AML patients has been somewhat improved, yet the long-term survival rate remained quite low^3^. The study found that the five-year survival rate of AML was only 20–30%^4,5^. Therefore, it is of great necessity to evaluate the burden of AML in order to better understand its significance for public health.

The global tobacco epidemic remains a major public health issue. In 2019, globally, there were 1.14 billion smokers, with the age-standardized prevalence rate being 32.7% among the male population and 6.62% among the female population^6^. Tobacco was a main risk factor for the occurrence and development of AML^7^. A study had found that the risk of AML increased for both former smokers and current smokers^8^. There were also studies indicating that tobacco was associated with the prognosis of patients with AML. Among the AML population, the survival rate of smoking patients was lower compared with that of non-smoking patients^7^. Smoking is one of the most easily modifiable risk factors, and multiple countries have introduced policies to reduce tobacco consumption^9^. However, the increase in the number of smokers driven by population growth and unequal distribution of resources remains an issue that requires attention at the present stage^10^.

The Global Burden of Disease (GBD) 2021 provides comprehensive data on AML attributable to tobacco, which deserves timely attention and analysis. Therefore, in this study, we explored and investigated the burden of AML attributable to tobacco in 204 countries and regions from 1990 to 2021.

## METHODS

### Data source

This is a secondary data analysis of the GBD dataset. Data on the burden of AML attributable to tobacco were obtained from the Global Health Data Exchange (GHDx) query tool, and a secondary dataset analysis approach was employed to address the research objectives. The GBD data include the number of incidences, prevalence, deaths, disability adjusted life years (DALYs) each year, as well as the corresponding age-standardized rates. The number and age-standardized rates (ASR) of AML attributable to tobacco deaths and DALYs from 1990 to 2021 were extracted from the GBD 2021 study, categorized by sociodemographic index (SDI) and age, region, and country.

### Related definition

The classification of AML in the GBD 2021 study was based on the International Classification of Diseases, Ninth Revision (ICD-9) and Tenth Revision (ICD-10). In detail, incident AML cases were ascertained using either ICD-9 codes C92.0-C92.02, C92.3-C92.62, C93.0-C93.02, C94.0-C94.02, C94.2-C94.22, C94.4-C94.5 or ICD-10 codes 205.0–205.02, 205.2–205.32, 206.0–206.02, 207.0–207.02, 207.2–207.82^11^. In the GBD 2021 study, tobacco includes multiple modalities, including current or past usage of any smoked tobacco product, current use of any chewing tobacco product, and the average daily exposure to air particulate matter from second-hand smoke. The SDI was calculated from national per capita income, total fertility rate in those <25 years, and average schooling in those ≥15 years. The SDI regions were divided into high, high-middle regions, middle, low-middle, and low.

### Statistical analysis

In this study, the age-standardized rate (ASR) per 100000 people was calculated. The results were reported with corresponding 95% uncertainty interval (UI). Consistent with the standard analytical protocol of the GBD study, the 95% UI was defined as the interval spanning the 2.5th to 97.5th percentiles of sorted results from 1000 iterative sampling simulations.

The estimated annual percentage change (EAPC) in ASR was calculated to characterize the average trend over a given period. EAPC and its 95% confidence interval (CI) were derived from a linear regression model. A positive EAPC, along with its corresponding 95% CI, indicates an upward trend in ASR, while a negative EAPC and its corresponding 95% CI suggest a downward trend in ASR^12^.

The age-period-cohort (APC) model was used to explore the temporal trends of AML attributable to tobacco in terms of age, period, and cohort effects globally from 1990 to 2021. To represent the age effect, the model fitted a longitudinal age curve reflecting the age-related natural history and adjusted the longitudinal age-specific rates in the reference cohort to accommodate the period deviation. The period/cohort effects are expressed by the risk ratio (RR), and its calculation method is the ratio of the age-specific rate in each period/cohort to the age-specific rate of the reference period/cohort^13^. This study was statistically examined using the R-based APC model analysis toolkit developed by IHME.

The Bayesian age-period-cohort (BAPC) model and the integrated nested Laplace approximation method were used to predict the ASDR and the age-standardized DALYs rate of AML attributable to tobacco globally from 2022 to 2050 based on gender. This study used the statistical software R version 4.4.3 for statistical analysis and visualization, and p<0.05 was considered to a statistically significant difference.

## RESULTS

### Global level

From 1990 to 2021, the burden of AML attributable to tobacco globally showed downward trends (Table 1). In 2021, the number of deaths of AML attributable to tobacco was14938 (95% UI: 5250–26001), compared to 8091 (95% UI: 3046–13141) deaths in 1990. In 2021, the number of DALYs of AML attributable to tobacco was 308708 (95% UI: 112920–524924), compared to194076 (95% UI: 75305–313550) DALYs in 1990. From 1990 to 2021, the ASDR decreased annually by -0.55 (95% CI: -0.71 – -0.4). Over the study period, the age-standardized DALY rate also showed a decline, with an EAPC of -0.93 (95% CI: -1.08 – -0.78) (Table 1).

**Table 1:** Global and regional deaths, DALYs, and estimated annual percentage change for acute myeloid leukemia attributable to tobacco, 1990–2021, based on Global Burden of Disease 2021 data

*Location*	*1990*			*2021*			*EAPC (95% CI)*
	*Number (95% UI)*	*ASR (95% UI)*		*Number (95% UI)*		*ASR (95% UI)*	
**Deaths**							
**Global**	8091 (3046–13141)	0.214 (0.08–0.35)		14938 (5250–26001)		0.176 (0.062–0.307)	-0.55 (-0.71 – -0.4)
**SDI regions**							
High	5106 (1897–8344)	0.453 (0.169–0.74)		8552 (2761–15149)		0.381 (0.125–0.669)	-0.46 (-0.64–0.27)
High-middle	1662 (648–2719)	0.169 (0.065–0.277)		3373 (1279–5650)		0.169 (0.064–0.283)	0.11 (-0.05–0.26)
Middle	920 (354–1593)	0.097 (0.037–0.17)		2097 (782–3594)		0.081 (0.03–0.14)	-0.72 (-0.8 – -0.63)
Low-middle	340 (120–672)	0.062 (0.022–0.124)		793 (283–1444)		0.059 (0.021–0.108)	-0.09 (-0.16 – -0.03)
Low	53 (15–104)	0.026 (0.007–0.051)		107 (34–199)		0.023 (0.007–0.044)	-0.42 (-0.52 – -0.32)
**Continents**							
Africa	132 (43–288)	0.051 (0.016–0.115)		351 (114–741)		0.059 (0.019–0.126)	0.76 (0.61–0.9)
America	2741 (999–4548)	0.448 (0.163–0.744)		4685 (1477–8406)		0.34 (0.107–0.61)	–0.87 (-1.09 – -0.65)
Asia	2132 (836–3635)	0.115 (0.044–0.196)		4819 (1760–8015)		0.099 (0.036–0.166)	-0.51 (-0.64 – -0.38)
Europe	3072 (1181–4919)	0.291 (0.112–0.467)		5057 (1763–8736)		0.3 (0.106–0.517)	0.3 (0.17–0.43)
**WHO regions**							
African	47 (15–88)	0.024 (0.008–0.046)		81 (27–149)		0.018 (0.006–0.034)	-0.93 (-0.97 – -0.89)
Eastern Mediterranean	264 (86–547)	0.156 (0.051–0.334)		681 (231–1330)		0.167 (0.056–0.33)	0.43 (0.31–0.54)
European	3112 (1197–4985)	0.286 (0.11–0.458)		5133 (1791–8869)		0.294 (0.104–0.506)	0.29 (0.16–0.43)
Americas	2741 (999–4548)	0.448 (0.163–0.744)		4685 (1477–8406)		0.34 (0.107–0.61)	-0.87 (-1.09 – -0.65)
South-East Asia	322 (107–596)	0.054 (0.018–0.1)		768 (260–1350)		0.047 (0.016–0.084)	-0.6 (-0.66 – -0.53)
Western Pacific	1557 (617–2640)	0.145 (0.056–0.246)		3454 (1260–5873)		0.122 (0.044–0.205)	-0.59 (-0.73 – -0.44)
**GBD regions**							
Andean Latin America	5 (2–10)	0.028 (0.009–0.054)		16 (5–30)		0.027 (0.009–0.052)	0.27 (0.08–0.45)
Australasia	78 (27–134)	0.323 (0.109–0.555)		154 (44–309)		0.265 (0.078–0.527)	-0.86 (-1.14 – -0.57)
Caribbean	28 (10–50)	0.109 (0.039–0.197)		53 (18–100)		0.099 (0.033–0.186)	-0.21 (-0.34 – -0.08)
Central Asia	37 (14–63)	0.075 (0.029–0.13)		63 (25–105)		0.076 (0.029–0.126)	0.7 (0.46–0.93)
Central Europe	367 (143–605)	0.241 (0.094–0.399)		584 (207–988)		0.253 (0.091–0.426)	0.66 (0.45–0.88)
Central Latin America	36 (14–61)	0.045 (0.017–0.076)		74 (27–131)		0.03 (0.011–0.054)	-1.61 (-1.76 – -1.45)
Central Sub-Saharan Africa	2 (1–5)	0.011 (0.003–0.023)		5 (2–11)		0.01 (0.003–0.022)	-0.24 (-0.52–0.03)
East Asia	792 (287–1423)	0.098 (0.036–0.177)		2077 (772–3865)		0.095 (0.035–0.177)	-0.18 (-0.31 – -0.05)
Eastern Europe	278 (110–472)	0.098 (0.038–0.165)		392 (153–631)		0.111 (0.043–0.178)	0.53 (0.33–0.74)
Eastern Sub-Saharan Africa	6 (2–12)	0.008 (0.002–0.016)		11 (3–22)		0.006 (0.002–0.013)	-0.9 (-0.96 – -0.83)
High-income Asia Pacific	623 (262–984)	0.308 (0.129–0.486)		1128 (378–1984)		0.219 (0.075–0.38)	-0.97 (-1.19 – -0.74)
High-income North America	2418 (870–4028)	0.678 (0.246–1.124)		4048 (1262–7253)		0.584 (0.183–1.042)	-0.45 (-0.72 – -0.19)
North Africa and Middle East	438 (150–821)	0.274 (0.093–0.526)		1006 (346–1874)		0.239 (0.081–0.445)	-0.32 (-0.39 – -0.26)
Oceania	2 (1–4)	0.08 (0.023–0.153)		4(1–7)		0.053 (0.015–0.102)	-1.4 (-1.55 – -1.25)
South Asia	191 (61–363)	0.039 (0.012-0.075)	407 (138–719)			0.031 (0.01–0.054)	-0.96 (-1.03 – -0.89)
Southeast Asia	265 (90–472)	0.119 (0.041–0.21)	613 (215–1073)			0.105 (0.037–0.186)	-0.62 (-0.73 – -0.51)
Southern Latin America	69 (25–119)	0.147 (0.054–0.257)	116 (41–210)			0.132 (0.046–0.236)	-0.08 (-0.41–0.25)
Southern Sub-Saharan Africa	23 (7–43)	0.09 (0.029–0.175)	26 (9–48)			0.047 (0.016–0.089)	-2.12 (-2.25 – -1.99)
Tropical Latin America	192 (72–325)	0.221 (0.082–0.377)	391 (123–721)			0.156 (0.049–0.289)	-1.16 (-1.27 – -1.04)
Western Europe	2238 (844–3628)	0.374 (0.142–0.607)	3766 (1280–6670)			0.369 (0.129–0.649)	0.13 (-0.03–0.29)
Western Sub-Saharan Africa	2 (1–4)	0.003 (0.001–0.005)	4 (1–8)			0.002 (0.001–0.005)	-0.39 (-0.46 – -0.32)
**World Bank regions**							
High Income	783 (282–1280)	0.499 (0.18–0.816)	1202 (384–2219)			0.407 (0.13–0.746)	-0.59 (-0.7 – -0.47)
Middle Income	200 (65–370)	0.037 (0.012–0.07)	412 (140–728)			0.029 (0.01–0.052)	-0.95 (-1.02 – -0.87)
Low Income	26 (8–51)	0.033 (0.01–0.067)	54 (17–108)			0.027 (0.009–0.052)	-0.83 (-0.93 – -0.72)
**DALYs**							
**Global**	194076 (75305–313550)	4.833 (1.856–7.823)	308708 (112920–524924)			3.566 (1.299–6.084)	-0.93 (-1.08 – -0.78)
**SDI regions**							
High-middle	42894 (17115–70215)	4.179 (1.659–6.865)	74705 (28434–122644)			3.751 (1.428–6.173)	-0.29 (-0.43 – -0.15)
High	116003 (44246–186929)	10.562 (4.051–17.004)	161470 (54476–280596)			7.69 (2.639–13.251)	-0.93 (-1.12 – -0.74)
Low-middle	8873 (3113–16998)	1.419 (0.5–2.766)	19549 (7068–35306)			1.334 (0.48–2.428)	-0.15 (-0.2 – -0.1)
Low	1437 (407–2806)	0.611 (0.173–1.191)	2834 (908–5153)			0.533 (0.169–0.983)	-0.51 (-0.6 – -0.42)
Middle	24634 (9506–42400)	2.272 (0.877–3.879)	49814 (18727–85482)			1.819 (0.683–3.109)	-0.87 (-0.94 – -0.79)
**Continents**							
Africa	3503 (1172–7389)	1.184 (0.391–2.565)	9252 (3029–18640)			1.358 (0.441–2.826)	0.71 (0.57–0.85)
America	63776 (23959–103711)	10.404 (3.91–16.925)	93341 (30605–164902)			6.831 (2.251–12.052)	-1.37 (-1.59 – -1.16)
Asia	55386 (21541–94677)	2.647 (1.038–4.527)	107109 (40090–179456)			2.101 (0.783–3.513)	-0.79 (-0.9 – -0.67)
Europe	71056 (28001–112417)	6.862 (2.707–10.873)	98463 (35465–168027)			6.257 (2.28–10.602)	-0.13 (-0.27–0.01)
**WHO regions**							
African	1208 (401–2210)	0.53 (0.173–0.998)	2079 (687–3908)			0.397 (0.13–0.731)	-1.00 (-1.04 – -0.96)
Eastern Mediterranean	6968 (2311–14078)	3.71 (1.222–7.656)	17575 (6129–33720)			3.762 (1.287–7.322)	0.23 (0.12–0.34)
European	72138 (28463–114156)	6.727 (2.657–10.661)	100251 (36150–171036)			6.097 (2.222–10.339)	-0.14 (-0.29–0)
Americas	63776 (23959–103711)	10.404 (3.91–16.925)	93341 (30605–164902)			6.831 (2.251–12.052)	-1.37 (-1.59 – -1.16)
South-East Asia	8482 (2828–15399)	1.19 (0.396–2.194)	18119 (6163–31734)			1.002 (0.341–1.775)	-0.71 (-0.77 – -0.65)
Western Pacific	40263 (15667–68354)	3.374 (1.317–5.722)	74575 (28096–126866)			2.609 (0.982–4.442)	-0.86 (-0.99 – -0.73)
**GBD regions**							
Andean Latin America	140 (50–256)	0.652 (0.227–1.205)	385 (136–708)			0.635 (0.224–1.177)	0.27 (0.09–0.44)
Australasia	1705 (597–2870)	7.097 (2.499–11.951)	2761 (862–5367)			5.054 (1.628–9.666)	-1.33 (-1.62 – -1.05)
Caribbean	669 (246–1179)	2.535 (0.929–4.483)	1158 (407–2107)			2.153 (0.758–3.921)	-0.48 (-0.6 – -0.35)
Central Asia	1152 (457–1993)	2.261 (0.892–3.929)	1835 (726–3003)			2.033 (0.802–3.338)	0.17 (-0.03–0.37)
Central Europe	9437 (3766-15353)	6.149 (2.456–10.016)	12760 (4667–21232)			5.819 (2.158–9.632)	0.3 (0.08–0.51)
Central Latin America	995 (380–1659)	1.092 (0.418–1.836)	1809 (663–3072)			0.711 (0.261–1.213)	-1.73 (-1.86 – -1.61)
Central Sub-Saharan Africa	72 (21–139)	0.285 (0.084–0.564)			170 (52–349)	0.26 (0.08–0.539)	-0.17 (-0.42–0.09)
East Asia	21542 (7656–38791)	2.347 (0.85–4.25)			49136 (18163–90218)	2.197 (0.801–4.036)	-0.28 (-0.38 – -0.17)
Eastern Europe	7954 (3126–13376)	2.797 (1.095–4.7)			10049 (3993–16080)	2.933 (1.164–4.715)	0.14 (-0.05–0.33)
Eastern Sub-Saharan Africa	183 (51–354)	0.218 (0.061–0.423)			340 (96–672)	0.17 (0.048–0.337)	-0.86 (-0.92 – -0.81)
High-income Asia Pacific	15516 (6528–24576)	7.502 (3.156–11.873)			19809 (6753–34354)	4.4 (1.543–7.558)	-1.58 (-1.8 – -1.36)
High-income North America	55340 (20519–90244)	16.226 (6.054–26.392)			79278 (25449–140266)	11.839 (3.862–20.817)	-1.04 (-1.29 – -0.78)
North Africa and Middle East	11882 (4159–22077)	6.648 (2.297–12.439)			25349 (8886–47152)	5.405 (1.875–10.073)	-0.59 (-0.65 – -0.53)
Oceania	61 (17–112)	1.871 (0.528–3.432)			120 (32–229)	1.339 (0.361–2.504)	-1.12 (-1.24 – -1)
South Asia	4915 (1572–9356)	0.853 (0.274–1.625)			9409 (3295–16594)	0.641 (0.223–1.134)	-1.09 (-1.15 – -1.03)
Southeast Asia	6808 (2293–12248)	2.626 (0.894–4.673)			14885 (5068–25988)	2.259 (0.776–3.952)	-0.68 (-0.78 – -0.57)
Southern Latin America	1786 (660–3003)	3.778 (1.4–6.374)			2676 (971–4615)	3.123 (1.143–5.348)	-0.39 (-0.72 – -0.05)
Southern SubSaharan Africa	586 (196–1080)	2.064 (0.685–3.869)			705 (253-1287)	1.139 (0.4–2.107)	-1.97 (-2.08 – -1.86)
Tropical Latin America	4991 (1894–8355)	5.209 (1.97–8.838)			8282 (2687–15023)	3.221 (1.04–5.847)	-1.7 (-1.81 – -1.59)
Western Europe	48284 (18647–77271)	8.491 (3.307–13.572)			67673 (23912–117479)	7.285 (2.616–12.502)	-0.34 (-0.51 – -0.16)
Western Sub-Saharan Africa	57 (18–107)	0.062 (0.02–0.118)			120 (41–214)	0.056 (0.019–0.103)	-0.41 (-0.49 – -0.32)
**World Bank regions**							
High Income	16449 (6106–26701)	10.891 (4.074–17.687)			21267 (6919–38262)	7.633 (2.524–13.564)	-1.13 (-1.26 – -0.99)
Middle Income	5194 (1699–9598)	0.823 (0.266–1.516)			9685 (3404-16742)	0.621 (0.214–1.085)	-1.06 (-1.13 – -1)
Low Income	650 (189–1249)	0.735 (0.216–1.435)			1283 (398–2596)	0.558 (0.176–1.116)	-0.94 (-1.01 – -0.87)

DALYs: disability-adjusted life years. ASR: age-standardized rate. SDI: social demographic index. UI: uncertainty interval. CI: confidence interval.

### Regional level

Among SDI regions, from 1990 to 2021, only the ASDR of AML attributable to tobacco in the high-middle SDI regions maintained a stable trend, while the ASDRs of AML attributable to tobacco in the other regions all showed a downward trend (Table 1). The high SDI regions had the highest ASDR [0.381 (95% UI: 0.125–0.669) per 10000 people] and age-standardized DALYs rate [7.69 (95% UI: 2.639–13.251) per 100000 people] for AML attributable to tobacco in 2021. The lowest of those was found in low SDI regions with rates of 0.023 (95% UI: 0.007–0.044) and 0.533 (95% UI: 0.169–0.983), respectively. Low-middle SDI regions had the slightest decrease in ASDR and age-standardized DALYs rate of AML attributable to tobacco from 1990 to 2021. The region with the greatest decrease in ASDR of AML attributable to tobacco from 1990 to 2021 was the middle SDI regions (EAPC= -0.72; 95% CI: -0.8 – -0.63), while that of age-standardized DALYs rate was high SDI regions (EAPC= -0.93; 95% CI: -1.12 – -0.74).

Among the four continents, America had the highest age-standardized DALYs rate of AML attributable to tobacco in 2021, with rates of 0.34 (95% UI: 0.107–0.61) and 6.831 (95% UI: 2.251–12.052), respectively (Table 1). The lowest of those was found in Africa with rates of 0.059 (95% UI: 0.019–0.126) and 1.358 (95% UI: 0.441–2.826), respectively. At the same time, America experienced the greatest decrease in the ASDR and the age-standardized DALYs rate from 1990 to 2021, and Africa experienced the greatest increase in those.

Similarly, among WHO regions, the African region had the lowest ASDR and age-standardized DALYs rate of AML attributable to tobacco in 2021, while the Region of the Americas had the highest corresponding rates. Among GBD regions, High-income North America had the highest ASDR and age-standardized DALYs rate, with rates of 0.584 (95% UI: 0.183–1.042) and 11.839 (95% UI: 3.862– 20.817), respectively. The lowest of those was found in Western Sub-Saharan Africa, with rates of 0.002 (95% UI: 0.001–0.005) and 0.056 (95% UI: 0.019–0.103), respectively. Central Asia had the highest increase in ASDR (EAPC=0.7; 95% CI: 0.46–0.93), and Central Europe had the highest increase in age-standardized DALYs rate (EAPC= 0.3; 95% CI: 0.08–0.51). Southern Sub-Saharan Africa reached the greatest decrease in ASDR and age-standardized DALYs rate (Table 1). Among World Bank regions, high-income regions had the highest ASDR [0.407 (95% UI: 0.13–0.746) per 100000 people] and age-standardized DALYs rate [7.633 (95% UI: 2.524–13.564) per 100000 people] for AML attributable to tobacco in 2021. Low-income regions had the lowest of those (Table 1).

### National level

**Figure 1:**
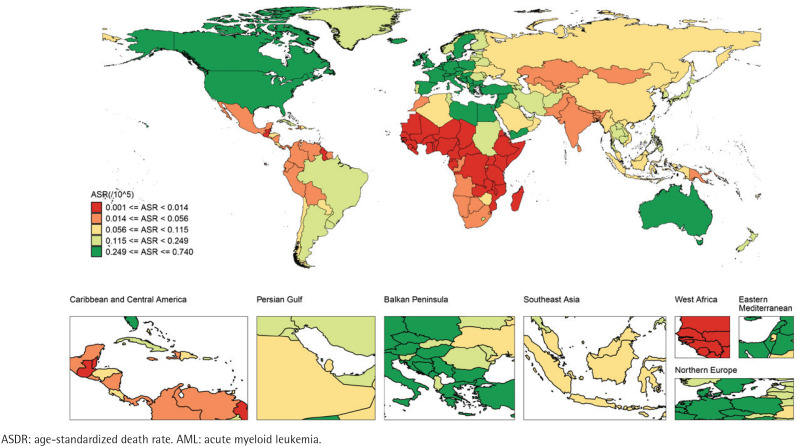
The ASDR of AML attributable to tobacco in 204 countries and territories in 2021, based on Global Burden of Disease 2021 data

**Figure 2:**
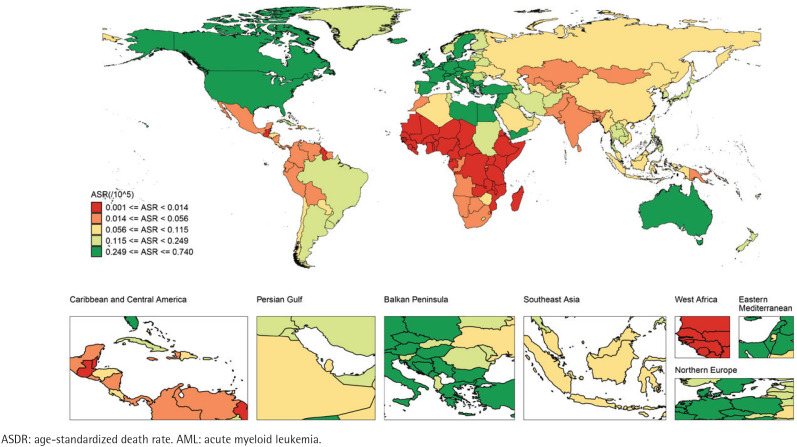
The age-standardized DALYs rate of AML attributable to tobacco in 204 countries and territories in 2021, based on Global Burden of Disease 2021 data

**Figure 3:**
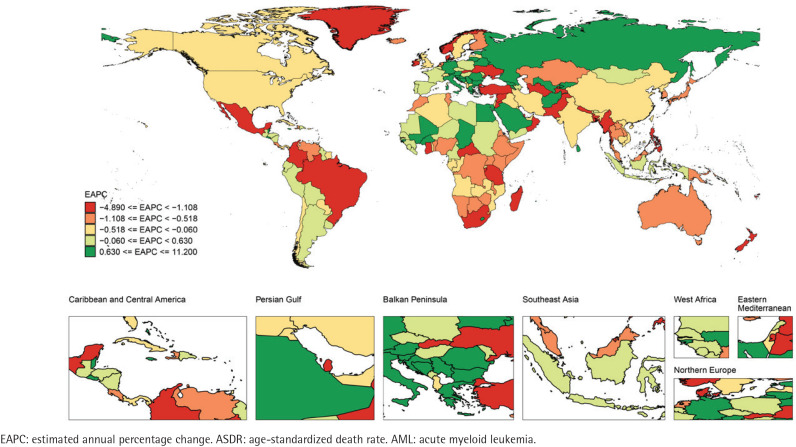
The EAPC of ASDR of AML attributable to tobacco in 204 countries and territories in 2021, based on Global Burden of Disease 2021 data

**Figure 4:**
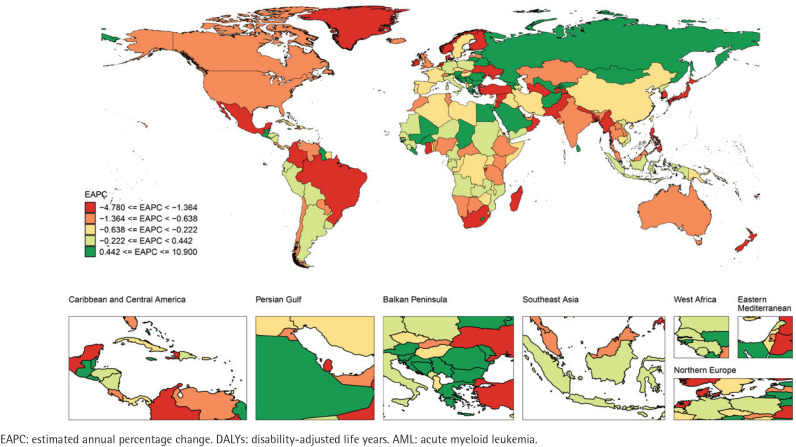
The EAPC of age-standardized DALYs rate of AML attributable to tobacco in 204 countries and territories in 2021, based on Global Burden of Disease 2021 data

At national levels, in 2021, the highest ASDR of AML attributable to tobacco was found in Monaco, with rate of 0.74 (95% UI: 0.185–1.536), and the lowest ASDR was found in Guinea and Sao Tome and Principe (0.001; 95% UI: 0–0.002) (Figures 1 and 2; and Supplementary file Table S1). Cyprus reached the highest age-standardized DALYs rate (9.833; 95% UI: 3.187–17.267) of AML attributable to tobacco in 2021, and Guinea had the lowest of this (0.025; 95% UI: 0.008–0.055). Northern Mariana Islands had the greatest decrease in ASDR (EAPC= -4.89; 95% CI: -5.29 – -4.49) and age-standardized DALYs rate (EAPC= -4.78; 95% CI: -5.16 – -4.41). The highest increase of those was found in Georgia, with EAPCs of 4 (95% CI: 3–5.01), and 3.59 (95% CI: 2.62–4.56), respectively (Figures 3 and 4; and Supplementary file Table S1).

### Sex and age level

Supplementary file Figure 1 shows the number of deaths and DALYs of AML attributable to tobacco in different age groups under different genders. The age group of 70–74 years had the largest number of male and female deaths, which were 2340 and 542, respectively. The age group with the highest number of DALYs was the 70–74 age group, regardless of sex, with 10954 females and 47356 males. The burden of AML attributable to tobacco showed a downward trend after the age of 75 years. The burden of AML attributable to tobacco showed a significant difference between males and females. At each age group, the burden of AML attributable to tobacco in males was significantly greater than that in females. When stratified by SDI, both males and females had the highest death and DALYs in the high SDI regions (Supplementary file Figure 2).

### Attribution of tobacco among SDI regions

Supplementary file Figure 3 showed the attribution of tobacco to AML, among the risk factors of AML, tobacco contributed to 11.5% of global AML deaths, with significant variation in the tobacco-attributable proportion of AML across SDI regions. High-SDI regions had the highest attributable share (18.1%), and low SDI regions had the lowest share at 1.8%. In terms of AML DALYs, tobacco shared at 7.5% in global. High SDI regions had the highest share at 15.6%, and low SDI regions had the lowest share at 0.9%.

### APC model

For the age effect, AML attributable to tobacco mortality rose mildly before age 40, then accelerated sharply, while DALYs peaked at age 30 and declined thereafter. For the period effect, the RR of tobacco-attributable AML mortality decreased continuously. For the cohort effect, risk decreased gently in birth cohorts prior to 2005–2010, and rapidly in subsequent cohorts. For the cohorts born before 1995–2000, the period RR of DALYs of AML attributable to tobacco decreased, while for those born after 1995–2000, it gradually increased with the increasing birth year. For the population born after 2005–2010, the period RR presented as a low downward trend, while for the population born after 2015–2020, the RR showed a rapid downward trend. The cohort RR of death of AML attributable to tobacco showed a rapid downward trend, and a slow downward trend was also found in DALYs from 1990 to 2021 (Supplementary file Figure 4).

### Future forecasts

Supplementary file Figure 5 showed the predicted results of the burden of AML attributable to tobacco for approximately three decades. Overall, in the next 30 years, for AML attributable to tobacco, both the ASDR and the age-standardized DALYs rate will show a downward trend regardless of gender. The ASDR for males decreases from 0.3 per 100000 people in 2021 to 0.24 per 100000 people, while for females, it decreases from 0.07 per 100000 people to 0.03 per 100000 people. The age-standardized DALY rate for males decreases from 6.51 per 100000 people in 2021 to 5.24 per 100000 people, while for females, it decreases from 1.45 per 100000 people to 0.61 per 100000 people.

## DISCUSSION

This study represents the latest assessment and research on the spatial and temporal trends of the burden of AML attributable to tobacco. The study had found that over the past 30 years, globally, the number of deaths and the DALYs of AML attributable to tobacco had increased, but both the ASDR and the age-standardized DALY rate of AML attributable to tobacco had shown a downward trend. WHO report showed Tobacco use declined from 2000 to 2030^14^, partially explaining the global drop in the burden of AML attributable to tobacco. Meanwhile, global population growth and aging have increased smoking-related disease burden^9^.

Tobacco contains a variety of carcinogenic substances, such as polycyclic aromatic hydrocarbons, nitrosamines, aromatic amines, etc.^15^. Benzo(a) pyrene, one of the polycyclic aromatic hydrocarbons, is metabolized in the body into benzo(a)pyrene-7,8-diol-9,10-epoxide which can bind to DNA, leading to DNA damage^16^. If these damages cannot be repaired in a timely manner, they may cause gene mutations, chromosomal aberrations etc., and further affect the normal function of hematopoietic stem cells, promoting their transformation into leukemia cells. Besides, related studies have found that nitrosamines in tobacco can block the targeting of centrosomes by p53, leading to chromosomal instability and thereby increasing tumor progression^17^. Studies have found that the activation of P53 can lead to significant regression of AML tumors through systemic immune responses^18^.

At the district level, this study found significant differences between districts. Whether in 1990 or 2021, the high SDI regions had the largest number of deaths and DALYs of AML attributable to tobacco, and their corresponding age-standardized rates were also the highest. A study found that among World Bank regions, upper middle-income regions and high-income regions had higher smoking prevalence, with rates of 236.570 per 100000 people, and 258.022 per 100000 people, respectively^19^. A study on risk factors in the GBD 2021 found that among all level 3 risk factors, smoking ranked first in high SDI regions^20^. It is obvious that economic factors affect tobacco consumption, and correspondingly, the burden of AML attributable to tobacco is heavier in high SDI regions^21^. Among 204 countries, the burden of AML attributable to tobacco showed a downward trend in most countries. This situation was attributed to the tobacco control policies implemented in these countries. For example, Brazil has implemented the MPOWER tobacco control measures, and its smoking rate has decreased by 35% since 2010^22^. Singapore has adopted a long-term, multi-pronged approach, including public education, smoking cessation measures, legislation, and taxation, to reduce tobacco use among Singaporeans. These efforts have led to a steady decline in the smoking rate among the Singaporean population over the years, dropping from 13.9% in 2010 to 11.8% in 2017 and further to 10.1% in 2020^23^.

This study also found that the burden of AML attributable to tobacco on the male population was significantly higher than that on the female population. Part of this situation may relate to the level of tobacco exposure. A study based on the American population found that 35% of men and 30% of women were exposed to environmental tobacco smoke, and 13% of men and 6% of women were current smokers^24^. Greater tobacco exposure means that men have more opportunities to be exposed to carcinogenic substances in tobacco, such as polycyclic aromatic hydrocarbons and nitrosamines^25^. These substances can damage the DNA of hematopoietic stem cells through various mechanisms, exacerbating AML. Part of it may also be related to occupational exposure. A national cross-sectional telephone survey study of Australian workers aged 18–65 years found that male respondents were more likely to be exposed to a variety of carcinogens^26^. Dust and chemical substances in these occupational environments interact with harmful components in tobacco, potentially increasing the risk of AML. Therefore, to reduce the burden of AML attributable to tobacco in men, it is essential to strengthen tobacco control, enhance occupational protection, and promote targeted health education.

This study showed that the burden of AML attributable to tobacco in the elderly population was significantly higher than that in the younger population. This condition was associated with long-term exposure to tobacco. A meta-analysis found that there was a positive correlation between the intensity of smoking, the duration of smoking, and the risk of AML^27^. In addition, it is related to declines in physical function and repair capacity among the elderly, as well as to the presence of multiple comorbidities. Multiple studies have found that the age of first smoking for most smokers is before the age of 20 years^28,29^. Arancini et al.^30^ conducted a study on 15874 smokers and found that compared with elderly smokers, young smokers are more likely to quit smoking. Although the burden of AML attributable to tobacco is mainly concentrated among the elderly population, tobacco control and smoking cessation programs and policies focusing on young people are effective solutions for reducing smoking rates among all populations. Therefore, it is necessary to formulate and promote diversified tobacco control strategies specifically targeting young people to achieve the optimal benefits.

In this study, it was found that the period from 2005 to 2010 was a special time. After this period, the period RR of deaths dropped rapidly, and the period RR of DALYs showed a downward trend. This situation cannot be separated from the implementation of smoking cessation policies and the emergence of new drugs for treating AML. The World Health Organization Framework Convention on Tobacco Control officially came into force on 27 February 2005^31^. During the period from 2005 to 2010, most countries formulated or revised their national tobacco control policies in accordance with the requirements of the Convention^32,33^. Between 2005 and 2010, the U.S. Food and Drug Administration approved two DNA methyltransferase inhibitors (azacitidine and decitabine) for the treatment of AML^34^. A meta-analysis indicated that the reported remission rate of AML for decitabine monotherapy was 40%^35^. Compared with the placebo group, oral azacitidine significantly prolonged the median overall survival and the median time to recurrence^36^.

### Limitations

This study has certain limitations. Firstly, although the GBD 2021 database provides comprehensive data support, there may be biases in the data collection process. The integrity and accuracy of data in some regions are less than ideal, which affects the reliability of the results. The calculation of disease burden indicators relies on model assumptions and parameter estimations. Even after rigorous verification, uncertainties remain. Secondly, although predictions have been made regarding deaths and DALYs, these predictions may be affected by uncontrollable factors, such as changes in health policies and the development of medical technologies, leading to discrepancies between the predictions and the actual figures. Thirdly, given the ecological modeling design, causal inference cannot be established. Additionally, tobacco-attributable estimates were not adjusted for other risk factors or potential confounders, which may have influenced the validity of the findings.

## CONCLUSIONS

This study revealed the spatial and temporal trends of the burden of AML attributable to tobacco. It was observed that globally, from 1990 to 2021, the burden of AML attributable to tobacco had been on a downward trend. The burden of AML attributable to tobacco was particularly prominent in high SDI regions, high-income regions, the male population, and the elderly population. Therefore, sustained tobacco control efforts, combined with population-specific interventions, may help alleviate the potential impact of tobacco on AML risk.

## Supplementary Material



## Data Availability

The data supporting this research are available from the following source: http://ghdx.healthdata.org
