# Supplementary material for: Global, regional, and national burden of acute myeloid leukemia attributable to tobacco from 1990 to 2021, with future forecasts to 2050: A secondary dataset analysis of the Global Burden of Disease 2021 study
Source: Tob Induc Dis. 2026 Jul 20;24:10.18332/tid/218783. doi: 10.18332/tid/218783 (PMC13401255; doi:10.18332/tid/218783)
Supplement: Supplementary file 1 [file TID-24-118-s1.pdf]

TID
Tobacco Induced Diseases

Supplementary file

© 2026 Zhang J. et al.

DOI:
10.18332/tid/218783

The content has been provided by the author(s) and has not been reviewed, verified, or endorsed by European Publishing. It may not have undergone peer review. The views, opinions, and recommendations expressed are solely those of the author(s) and do not necessarily reflect the position of European Publishing. European Publishing accepts no responsibility or liability for any consequences arising from the use of, or reliance on, this content.

Supplementary Table S1 The global disease burden of acute myeloid leukemia attributable to tobacco in 204 countries and territories

location	1990		2021		EAPC (95% CI)
	Number (95% UI)	ASR (95% UI)	Number (95% UI)	Number (95% UI)	
**Deaths**					
Afghanistan	9(2-23)	0.135(0.028-0.357)	13(3-30)	0.156(0.039-0.375)	0.67(0.41to0.93)
Albania	5(2-10)	0.258(0.088-0.505)	10(3-21)	0.23(0.074-0.458)	-0.07(-0.19to0.05)
Algeria	13(4-29)	0.134(0.04-0.264)	34(9-75)	0.114(0.031-0.243)	-0.3(-0.42to-0.17)
American Samoa	0(0-0)	0.069(0.017-0.142)	0(0-0)	0.024(0.008-0.052)	-4.11(-4.56to-3.66)
Andorra	0(0-1)	0.726(0.241-1.416)	1(0-1)	0.392(0.112-0.833)	-1.76(-1.97to-1.56)
Angola	1(0-2)	0.026(0.006-0.06)	3(1-6)	0.023(0.006-0.054)	-0.28(-0.51to-0.05)
Antigua and Barbuda	0(0-0)	0.075(0.022-0.146)	0(0-0)	0.094(0.029-0.181)	0.88(0.61to1.14)
Argentina	55(20-95)	0.167(0.06-0.292)	90(31-163)	0.158(0.055-0.284)	0.14(-0.23to0.5)
Armenia	3(1-6)	0.114(0.047-0.196)	8(4-13)	0.187(0.08-0.291)	2.2(1.53to2.88)
Australia	63(21-108)	0.311(0.106-0.542)	136(39-275)	0.278(0.081-0.555)	-0.62(-1.01to-0.24)
Austria	29(10-52)	0.243(0.087-0.424)	65(21-116)	0.342(0.113-0.6)	1.67(1.31to2.02)
Azerbaijan	6(2-12)	0.109(0.041-0.225)	12(4-27)	0.115(0.038-0.247)	0.53(0.32to0.74)
Bahamas	0(0-0)	0.045(0.012-0.097)	0(0-0)	0.058(0.017-0.115)	1.41(1.13to1.68)
Bahrain	1(0-1)	0.458(0.125-0.905)	2(1-5)	0.3(0.087-0.608)	-1.87(-2.09to-1.65)
Bangladesh	23(7-46)	0.053(0.015-0.11)	48(15-96)	0.038(0.012-0.073)	-1.24(-1.37to-1.11)
Barbados	0(0-0)	0.053(0.017-0.097)	0(0-1)	0.061(0.018-0.124)	0.52(0.22to0.83)
Belarus	14(5-26)	0.107(0.039-0.2)	29(12-50)	0.182(0.073-0.315)	1.75(1.36to2.14)
Belgium	75(28-121)	0.47(0.179-0.761)	120(40-213)	0.481(0.162-0.852)	0.27(-0.07to0.61)
Belize	0(0-0)	0.006(0.002-0.011)	0(0-0)	0.012(0.004-0.022)	2.29(1.55to3.04)
Benin	0(0-0)	0.002(0.001-0.005)	0(0-0)	0.002(0.001-0.004)	-0.97(-1.21to-0.73)
Bermuda	0(0-0)	0.126(0.038-0.265)	0(0-0)	0.098(0.03-0.193)	-0.31(-0.6to-0.02)
Bhutan	0(0-0)	0.04(0.009-0.104)	0(0-1)	0.042(0.011-0.104)	0.12(0.08to0.15)

Bolivia (Plurinational State of)	1(0-3)	0.042(0.013-0.096)	3(1-7)	0.039(0.011-0.083)	0.1(-0.01to0.2)
Bosnia and Herzegovina	9(3-17)	0.23(0.083-0.422)	18(6-34)	0.275(0.093-0.515)	1.03(0.75to1.31)
Botswana	0(0-1)	0.067(0.02-0.152)	1(0-1)	0.056(0.017-0.112)	-0.76(-0.89to-0.64)
Brazil	189(70-322)	0.223(0.082-0.381)	385(120-711)	0.157(0.049-0.292)	-1.17(-1.29to-1.06)
Brunei Darussalam	0(0-1)	0.429(0.162-0.79)	1(0-1)	0.237(0.069-0.446)	-1.38(-1.58to-1.19)
Bulgaria	26(10-43)	0.209(0.081-0.343)	46(16-80)	0.328(0.119-0.564)	2.21(1.86to2.56)
Burkina Faso	0(0-0)	0.001(0-0.003)	0(0-0)	0.002(0.001-0.004)	0.75(0.7to0.79)
Burundi	0(0-0)	0.009(0.002-0.019)	0(0-1)	0.005(0.001-0.012)	-1.6(-1.97to-1.22)
Cabo Verde	0(0-0)	0.008(0.003-0.022)	0(0-0)	0.009(0.003-0.02)	-0.28(-0.66to0.11)
Cambodia	10(3-20)	0.251(0.073-0.508)	25(9-45)	0.231(0.084-0.421)	-0.44(-0.5to-0.37)
Cameroon	0(0-0)	0.003(0.001-0.006)	0(0-1)	0.003(0.001-0.006)	0.15(-0.03to0.32)
Canada	180(65-299)	0.546(0.197-0.909)	331(103-607)	0.426(0.133-0.778)	-0.26(-0.45to-0.07)
Central African Republic	0(0-0)	0.013(0.004-0.03)	0(0-0)	0.009(0.003-0.018)	-1.29(-1.46to-1.13)
Chad	0(0-0)	0.002(0.001-0.005)	0(0-0)	0.003(0.001-0.007)	0.82(0.58to1.06)
Chile	7(3-13)	0.072(0.026-0.129)	15(5-27)	0.059(0.021-0.107)	-0.27(-0.55to0)
China	746(259-1353)	0.096(0.034-0.176)	1942(704-3621)	0.092(0.033-0.172)	-0.24(-0.35to-0.14)
Colombia	7(3-12)	0.039(0.015-0.07)	15(5-30)	0.028(0.009-0.054)	-1.74(-1.94to-1.54)
Comoros	0(0-0)	0.011(0.003-0.024)	0(0-0)	0.009(0.003-0.019)	-1.1(-1.2to-0.99)
Congo	0(0-0)	0.017(0.005-0.038)	0(0-1)	0.015(0.005-0.031)	-0.3(-0.45to-0.15)
Cook Islands	0(0-0)	0.046(0.011-0.106)	0(0-0)	0.032(0.008-0.073)	-1.19(-1.22to-1.15)
Costa Rica	2(1-4)	0.129(0.045-0.235)	6(2-11)	0.109(0.036-0.2)	-0.6(-0.84to-0.36)
Croatia	19(7-32)	0.322(0.117-0.526)	38(14-64)	0.397(0.144-0.667)	1.41(1.16to1.66)
Cuba	16(6-28)	0.158(0.058-0.278)	27(10-47)	0.137(0.049-0.239)	-0.33(-0.5to-0.17)
Cyprus	5(2-10)	0.656(0.24-1.246)	11(3-19)	0.486(0.15-0.875)	-0.52(-0.69to-0.35)
Czechia	49(17-83)	0.342(0.122-0.58)	64(22-113)	0.277(0.097-0.486)	0.09(-0.25to0.43)
Côte d'Ivoire	0(0-0)	0.006(0.002-0.014)	0(0-1)	0.005(0.001-0.011)	-1.07(-1.38to-0.75)

Democratic People's Republic of Korea	13(5-26)	0.08(0.029-0.16)	25(8-53)	0.075(0.026-0.156)	-0.24(-0.32to-0.16)
Democratic Republic of the Congo	1(0-2)	0.007(0.002-0.014)	2(1-5)	0.005(0.002-0.011)	-0.72(-0.99to-0.44)
Denmark	70(28-111)	0.816(0.323-1.298)	72(25-126)	0.539(0.185-0.949)	-1.26(-1.52to-0.99)
Djibouti	0(0-0)	0.018(0.004-0.046)	0(0-0)	0.016(0.004-0.035)	-0.21(-0.34to-0.08)
Dominica	0(0-0)	0.031(0.01-0.064)	0(0-0)	0.023(0.008-0.048)	-1(-1.06to-0.95)
Dominican Republic	3(1-8)	0.09(0.029-0.235)	8(3-17)	0.087(0.026-0.175)	0.15(-0.19to0.5)
Ecuador	2(1-4)	0.042(0.014-0.076)	5(2-10)	0.034(0.012-0.063)	0.27(-0.22to0.76)
Egypt	46(13-129)	0.185(0.05-0.598)	187(59-413)	0.335(0.103-0.812)	2.64(2.27to3)
El Salvador	1(0-2)	0.025(0.007-0.056)	2(1-4)	0.033(0.009-0.067)	1.08(0.94to1.21)
Equatorial Guinea	0(0-0)	0.01(0.003-0.02)	0(0-0)	0.009(0.003-0.02)	0.05(-0.2to0.29)
Eritrea	0(0-0)	0.006(0.002-0.016)	0(0-0)	0.006(0.001-0.015)	-0.57(-0.71to-0.43)
Estonia	2(1-3)	0.081(0.028-0.147)	5(2-8)	0.183(0.064-0.312)	2.77(1.72to3.84)
Eswatini	0(0-0)	0.023(0.008-0.048)	0(0-0)	0.018(0.006-0.039)	-0.63(-0.97to-0.29)
Ethiopia	2(0-4)	0.008(0.002-0.018)	3(1-7)	0.006(0.001-0.015)	-0.75(-1.01to-0.48)
Fiji	1(0-1)	0.208(0.062-0.395)	1(0-1)	0.105(0.03-0.217)	-2.28(-2.51to-2.05)
Finland	15(5-28)	0.201(0.067-0.378)	19(6-38)	0.139(0.043-0.27)	-1.09(-1.26to-0.92)
France	269(94-460)	0.313(0.111-0.54)	439(139-818)	0.28(0.092-0.509)	0.13(-0.15to0.41)
Gabon	0(0-0)	0.012(0.003-0.025)	0(0-0)	0.011(0.003-0.023)	-0.2(-0.28to-0.12)
Gambia	0(0-0)	0.003(0.001-0.007)	0(0-0)	0.002(0.001-0.005)	-1.44(-1.6to-1.28)
Georgia	7(3-12)	0.107(0.04-0.188)	12(5-20)	0.21(0.086-0.341)	4(3to5.01)
Germany	463(168-777)	0.357(0.128-0.603)	857(293-1510)	0.408(0.14-0.711)	0.68(0.56to0.81)
Ghana	0(0-1)	0.005(0.002-0.01)	0(0-1)	0.003(0.001-0.007)	-1.88(-2.23to-1.53)
Greece	80(32-125)	0.513(0.206-0.801)	182(68-300)	0.703(0.271-1.144)	1.07(0.85to1.29)
Greenland	0(0-0)	0.403(0.136-0.753)	0(0-0)	0.199(0.053-0.433)	-2.22(-2.36to-2.09)

Grenada	0(0-0)	0.016(0.005-0.032)	0(0-0)	0.02(0.007-0.035)	0.04(-0.52to0.6)
Guam	0(0-0)	0.058(0.019-0.131)	0(0-0)	0.064(0.021-0.127)	0.82(0.27to1.36)
Guatemala	0(0-1)	0.011(0.004-0.022)	1(1-3)	0.013(0.005-0.024)	0.21(-0.11to0.52)
Guinea	0(0-0)	0.001(0-0.002)	0(0-0)	0.001(0-0.002)	0.28(0.22to0.33)
Guinea-Bissau	0(0-0)	0.002(0.001-0.004)	0(0-0)	0.003(0.001-0.007)	2.28(2.08to2.48)
Guyana	0(0-0)	0.007(0.002-0.013)	0(0-0)	0.008(0.002-0.015)	0.63(0.36to0.9)
Haiti	1(0-1)	0.02(0.006-0.042)	1(0-2)	0.014(0.004-0.028)	-1.08(-1.16to-1)
Honduras	1(0-2)	0.054(0.017-0.116)	4(1-8)	0.062(0.017-0.141)	0.63(0.51to0.74)
Hungary	50(19-84)	0.333(0.127-0.557)	63(22-112)	0.314(0.11-0.562)	0(-0.22to0.22)
Iceland	1(0-2)	0.465(0.167-0.768)	2(1-4)	0.403(0.128-0.728)	-0.54(-0.66to-0.41)
India	126(40-246)	0.032(0.01-0.063)	304(102-549)	0.028(0.009-0.051)	-0.5(-0.57to-0.43)
Indonesia	62(19-120)	0.073(0.023-0.143)	183(63-330)	0.089(0.032-0.166)	0.5(0.26to0.75)
Iran (Islamic Republic of)	51(15-95)	0.203(0.058-0.395)	123(33-228)	0.168(0.044-0.315)	-0.31(-0.41to-0.21)
Iraq	20(6-55)	0.265(0.079-0.732)	54(16-132)	0.243(0.067-0.595)	-0.39(-0.51to-0.26)
Ireland	20(8-32)	0.46(0.174-0.743)	23(7-40)	0.268(0.088-0.477)	-1.6(-1.98to-1.22)
Israel	20(7-37)	0.41(0.146-0.744)	47(16-86)	0.363(0.124-0.656)	-0.1(-0.36to0.16)
Italy	261(92-439)	0.285(0.101-0.478)	558(190-980)	0.355(0.121-0.622)	0.91(0.64to1.18)
Jamaica	0(0-1)	0.024(0.008-0.045)	1(0-3)	0.048(0.015-0.096)	2.69(2.09to3.3)
Japan	526(208-832)	0.307(0.121-0.486)	889(305-1564)	0.218(0.077-0.377)	-0.95(-1.23to-0.67)
Jordan	11(4-19)	0.912(0.354-1.559)	41(15-73)	0.617(0.221-1.111)	-1.25(-1.41to-1.09)
Kazakhstan	11(3-20)	0.081(0.026-0.154)	10(4-19)	0.055(0.02-0.101)	-0.76(-1.23to-0.28)
Kenya	1(0-1)	0.008(0.002-0.018)	1(0-3)	0.006(0.002-0.012)	-0.87(-0.96to-0.79)
Kiribati	0(0-0)	0.072(0.015-0.157)	0(0-0)	0.09(0.018-0.208)	0.72(0.58to0.85)
Kuwait	1(0-2)	0.216(0.078-0.373)	4(1-8)	0.173(0.058-0.303)	-0.26(-0.98to0.47)
Kyrgyzstan	2(1-3)	0.058(0.022-0.101)	4(2-8)	0.087(0.035-0.144)	2.15(1.74to2.57)

Lao People's Democratic Republic	3(1-8)	0.186(0.054-0.392)	6(2-11)	0.153(0.054-0.272)	-0.64(-0.7to-0.59)
Latvia	6(2-10)	0.164(0.064-0.287)	5(2-9)	0.133(0.05-0.229)	-0.5(-0.84to-0.16)
Lebanon	10(4-20)	0.488(0.176-0.99)	31(11-55)	0.494(0.175-0.873)	0.46(0.22to0.69)
Lesotho	1(0-2)	0.094(0.03-0.218)	1(0-3)	0.127(0.039-0.268)	1.54(1.39to1.7)
Liberia	0(0-0)	0.002(0.001-0.004)	0(0-0)	0.002(0.001-0.004)	0.26(0.13to0.39)
Libya	5(2-13)	0.293(0.085-0.713)	12(4-26)	0.261(0.079-0.552)	-0.03(-0.17to0.12)
Lithuania	2(1-5)	0.055(0.019-0.102)	9(3-16)	0.153(0.056-0.268)	3.99(3.22to4.77)
Luxembourg	3(1-5)	0.509(0.177-0.914)	6(2-11)	0.527(0.15-0.971)	0.24(0to0.48)
Madagascar	0(0-1)	0.006(0.002-0.013)	0(0-1)	0.003(0.001-0.007)	-2.25(-2.52to-1.98)
Malawi	0(0-0)	0.003(0.001-0.007)	0(0-0)	0.003(0.001-0.006)	-0.55(-0.71to-0.39)
Malaysia	10(3-19)	0.119(0.037-0.223)	23(8-44)	0.091(0.031-0.173)	-1.04(-1.37to-0.71)
Maldives	0(0-0)	0.378(0.118-0.734)	1(0-1)	0.226(0.083-0.387)	-1.85(-1.93to-1.77)
Mali	0(0-0)	0.001(0-0.003)	0(0-0)	0.002(0.001-0.003)	0.93(0.86to1.01)
Malta	2(1-3)	0.38(0.135-0.66)	4(1-7)	0.358(0.122-0.65)	-0.06(-0.27to0.15)
Marshall Islands	0(0-0)	0.087(0.02-0.202)	0(0-0)	0.104(0.025-0.251)	0.82(0.7to0.95)
Mauritania	0(0-0)	0.003(0.001-0.007)	0(0-0)	0.003(0.001-0.006)	0.21(0.12to0.29)
Mauritius	0(0-0)	0.01(0.004-0.018)	1(0-1)	0.032(0.012-0.054)	11.2(5.44to17.27)
Mexico	20(7-33)	0.048(0.018-0.083)	32(12-54)	0.026(0.01-0.044)	-2.35(-2.52to-2.17)
Micronesia (Federated States of)	0(0-0)	0.083(0.024-0.182)	0(0-0)	0.08(0.023-0.161)	-0.06(-0.09to-0.03)
Monaco	1(0-1)	0.689(0.222-1.258)	1(0-2)	0.74(0.185-1.536)	0.35(0.11to0.58)
Mongolia	0(0-1)	0.045(0.016-0.086)	1(0-2)	0.05(0.016-0.091)	0.57(0.48to0.67)
Montenegro	2(1-4)	0.306(0.105-0.562)	3(1-6)	0.299(0.102-0.554)	0.02(-0.17to0.21)
Morocco	6(2-14)	0.041(0.011-0.105)	11(3-27)	0.033(0.009-0.081)	-0.65(-0.8to-0.5)
Mozambique	1(0-1)	0.009(0.003-0.019)	1(0-2)	0.008(0.002-0.016)	-0.16(-0.25to-0.08)

Myanmar	31(10-66)	0.157(0.054-0.321)	29(11-53)	0.069(0.025-0.128)	-2.9(-3.05to-2.76)
Namibia	0(0-0)	0.024(0.009-0.045)	0(0-0)	0.018(0.007-0.035)	-1.04(-1.3to-0.78)
Nauru	0(0-0)	0.105(0.029-0.231)	0(0-0)	0.078(0.021-0.168)	-1.09(-1.15to-1.03)
Nepal	3(1-5)	0.032(0.01-0.06)	5(2-9)	0.023(0.008-0.043)	-1.32(-1.45to-1.19)
Netherlands	113(42-186)	0.552(0.205-0.91)	153(50-280)	0.395(0.128-0.716)	-0.85(-1.03to-0.67)
New Zealand	15(5-27)	0.38(0.127-0.665)	18(6-34)	0.197(0.062-0.374)	-2.1(-2.43to-1.78)
Nicaragua	0(0-1)	0.025(0.008-0.052)	1(0-2)	0.024(0.008-0.044)	0.15(-0.04to0.34)
Niger	0(0-0)	0.002(0-0.003)	0(0-0)	0.002(0-0.004)	0.37(0.13to0.6)
Nigeria	1(0-2)	0.002(0.001-0.005)	1(0-3)	0.002(0.001-0.004)	-0.83(-0.87to-0.8)
Niue	0(0-0)	0.092(0.022-0.216)	0(0-0)	0.087(0.02-0.196)	-0.25(-0.31to-0.19)
North Macedonia	7(3-13)	0.39(0.145-0.746)	12(4-21)	0.347(0.105-0.63)	-0.38(-0.64to-0.13)
Northern Mariana Islands	0(0-0)	0.133(0.036-0.279)	0(0-0)	0.036(0.009-0.105)	-4.89(-5.29to-4.49)
Norway	24(9-42)	0.333(0.124-0.568)	20(6-38)	0.185(0.059-0.341)	-2.18(-2.39to-1.97)
Oman	1(0-2)	0.122(0.027-0.262)	1(0-3)	0.074(0.021-0.153)	-1.15(-1.36to-0.94)
Pakistan	40(12-79)	0.079(0.023-0.158)	50(16-91)	0.049(0.016-0.089)	-1.82(-2.02to-1.62)
Palau	0(0-0)	0.015(0.002-0.036)	0(0-0)	0.012(0.002-0.031)	-0.75(-0.82to-0.68)
Palestine	1(0-2)	0.116(0.035-0.235)	2(1-4)	0.092(0.028-0.186)	-1.12(-1.32to-0.92)
Panama	1(0-1)	0.054(0.018-0.1)	2(1-4)	0.051(0.017-0.099)	-0.39(-0.59to-0.2)
Papua New Guinea	1(0-1)	0.034(0.006-0.077)	2(0-4)	0.03(0.006-0.068)	-0.54(-0.65to-0.42)
Paraguay	3(1-5)	0.132(0.047-0.251)	6(2-12)	0.11(0.033-0.222)	-0.51(-0.66to-0.35)
Peru	2(1-4)	0.018(0.005-0.037)	7(2-15)	0.02(0.006-0.044)	0.31(0.13to0.49)
Philippines	53(18-95)	0.215(0.077-0.378)	96(34-172)	0.127(0.045-0.231)	-1.92(-2.13to-1.71)
Poland	124(46-208)	0.279(0.104-0.466)	194(68-340)	0.257(0.09-0.448)	0.32(-0.06to0.72)
Portugal	26(9-46)	0.179(0.059-0.319)	46(14-85)	0.176(0.057-0.315)	-0.02(-0.29to0.24)
Puerto Rico	5(2-11)	0.144(0.042-0.312)	11(3-24)	0.138(0.038-0.304)	-0.31(-0.51to-0.11)
Qatar	0(0-1)	0.249(0.064-0.534)	1(0-2)	0.101(0.03-0.192)	-3.13(-3.52to-2.74)

Republic of Korea	92(35-172)	0.319(0.121-0.605)	230(55-440)	0.242(0.058-0.462)	-0.84(-0.99to-0.69)
Republic of Moldova	9(3-16)	0.192(0.069-0.362)	12(4-19)	0.197(0.073-0.321)	0.63(0.07to1.18)
Romania	24(9-42)	0.086(0.031-0.149)	57(19-98)	0.153(0.051-0.26)	2.13(1.93to2.33)
Russian Federation	145(58-246)	0.078(0.031-0.133)	273(107-442)	0.112(0.044-0.182)	1.57(1.22to1.93)
Rwanda	0(0-1)	0.015(0.004-0.032)	1(0-2)	0.016(0.005-0.034)	-0.17(-0.32to-0.03)
Saint Kitts and Nevis	0(0-0)	0.01(0.003-0.018)	0(0-0)	0.011(0.003-0.023)	0.73(0.45to1.01)
Saint Lucia	0(0-0)	0.043(0.014-0.081)	0(0-0)	0.034(0.011-0.068)	-0.79(-1.07to-0.5)
Saint Vincent and the Grenadines	0(0-0)	0.012(0.004-0.023)	0(0-0)	0.021(0.007-0.039)	1.91(1.6to2.22)
Samoa	0(0-0)	0.247(0.065-0.509)	0(0-1)	0.245(0.073-0.499)	-0.22(-0.29to-0.14)
San Marino	0(0-0)	0.346(0.115-0.676)	0(0-0)	0.162(0.043-0.348)	-1.47(-1.8to-1.14)
Sao Tome and Principe	0(0-0)	0.001(0-0.002)	0(0-0)	0.001(0-0.002)	0.68(0.32to1.05)
Saudi Arabia	4(1-9)	0.061(0.016-0.156)	16(4-32)	0.083(0.021-0.168)	1.29(0.78to1.8)
Senegal	0(0-0)	0.004(0.001-0.008)	0(0-1)	0.004(0.001-0.01)	0.35(0.26to0.44)
Serbia	26(8-46)	0.239(0.078-0.425)	44(14-82)	0.262(0.085-0.483)	0.7(0.46to0.94)
Seychelles	0(0-0)	0.091(0.028-0.191)	0(0-0)	0.074(0.021-0.144)	-0.73(-0.92to-0.54)
Sierra Leone	0(0-0)	0.003(0.001-0.006)	0(0-0)	0.003(0.001-0.005)	-0.02(-0.16to0.13)
Singapore	5(2-9)	0.235(0.08-0.424)	8(3-16)	0.1(0.033-0.186)	-2.28(-2.68to-1.87)
Slovakia	14(5-29)	0.238(0.081-0.479)	15(4-31)	0.157(0.043-0.316)	-1.14(-1.22to-1.06)
Slovenia	5(2-9)	0.207(0.075-0.366)	11(3-20)	0.237(0.077-0.438)	1.2(0.88to1.52)
Solomon Islands	0(0-0)	0.103(0.024-0.218)	0(0-1)	0.102(0.025-0.201)	0.1(-0.02to0.22)
Somalia	0(0-0)	0.007(0.002-0.017)	0(0-1)	0.006(0.001-0.013)	-0.71(-0.78to-0.64)
South Africa	18(6-35)	0.091(0.028-0.184)	19(7-34)	0.043(0.014-0.078)	-2.52(-2.64to-2.39)
South Sudan	0(0-1)	0.009(0.002-0.021)	0(0-1)	0.007(0.002-0.016)	-0.9(-1.01to-0.79)
Spain	146(58-235)	0.265(0.106-0.426)	296(100-514)	0.289(0.1-0.498)	0.35(0.04to0.66)
Sri Lanka	1(0-4)	0.016(0.005-0.045)	4(1-9)	0.017(0.004-0.036)	1.42(0.73to2.12)

Sudan	19(5-41)	0.214(0.055-0.462)	39(9-97)	0.214(0.052-0.533)	-0.01(-0.06to0.04)
Suriname	0(0-0)	0.064(0.022-0.139)	0(0-1)	0.053(0.015-0.106)	-0.28(-0.56to0)
Sweden	55(20-99)	0.339(0.124-0.604)	84(26-157)	0.343(0.109-0.628)	-0.18(-0.35to0)
Switzerland	46(17-78)	0.435(0.16-0.736)	65(22-115)	0.325(0.112-0.572)	-0.27(-0.58to0.05)
Syrian Arab Republic	17(5-37)	0.346(0.108-0.776)	30(9-71)	0.255(0.074-0.602)	-1.21(-1.34to-1.08)
Taiwan	33(13-54)	0.221(0.088-0.359)	110(41-176)	0.255(0.097-0.41)	0.38(-0.09to0.85)
Tajikistan	3(1-7)	0.119(0.037-0.249)	4(1-8)	0.056(0.016-0.119)	-2.34(-2.58to-2.1)
Thailand	63(21-118)	0.208(0.068-0.392)	168(37-341)	0.156(0.034-0.318)	-1.05(-1.17to-0.93)
Timor-Leste	0(0-0)	0.077(0.018-0.185)	0(0-1)	0.066(0.019-0.144)	-0.52(-0.57to-0.46)
Togo	0(0-0)	0.011(0.003-0.027)	0(0-1)	0.01(0.002-0.022)	-0.42(-0.49to-0.35)
Tokelau	0(0-0)	0.1(0.021-0.24)	0(0-0)	0.079(0.019-0.176)	-0.76(-0.8to-0.72)
Tonga	0(0-0)	0.192(0.054-0.399)	0(0-0)	0.178(0.05-0.372)	-0.35(-0.45to-0.26)
Trinidad and Tobago	1(0-1)	0.075(0.026-0.137)	1(0-2)	0.066(0.022-0.13)	-0.27(-0.45to-0.08)
Tunisia	9(3-23)	0.19(0.06-0.478)	20(6-49)	0.153(0.048-0.378)	-0.83(-0.91to-0.75)
Turkey	200(73-343)	0.594(0.213-1.032)	341(121-602)	0.367(0.13-0.649)	-1.68(-1.86to-1.49)
Turkmenistan	2(1-3)	0.09(0.034-0.16)	3(1-5)	0.063(0.019-0.116)	-1.28(-1.48to-1.07)
Tuvalu	0(0-0)	0.086(0.018-0.194)	0(0-0)	0.082(0.023-0.167)	-0.01(-0.06to0.04)
Uganda	0(0-1)	0.004(0.001-0.008)	1(0-1)	0.004(0.001-0.008)	-0.69(-1.02to-0.37)
Ukraine	101(39-174)	0.139(0.053-0.241)	59(20-107)	0.077(0.026-0.138)	-2.49(-2.77to-2.21)
United Arab Emirates	1(0-2)	0.252(0.061-0.544)	4(1-8)	0.148(0.042-0.311)	-0.49(-0.92to-0.05)
United Kingdom	512(188-830)	0.537(0.198-0.87)	691(223-1254)	0.478(0.155-0.859)	-0.45(-0.63to-0.28)
United Republic of Tanzania	1(0-3)	0.012(0.003-0.025)	2(1-5)	0.009(0.002-0.018)	-1.38(-1.49to-1.26)
United States Virgin Islands	0(0-0)	0.061(0.016-0.137)	0(0-0)	0.031(0.008-0.076)	-2.06(-2.33to-1.79)
United States of America	2237(809-3728)	0.692(0.252-1.146)	3717(1151-6678)	0.603(0.188-1.079)	-0.46(-0.74to-0.17)
Uruguay	7(2-11)	0.165(0.06-0.284)	11(4-19)	0.2(0.071-0.349)	0.49(0.32to0.67)
Uzbekistan	2(1-5)	0.02(0.006-0.041)	8(3-15)	0.031(0.011-0.056)	1.53(1.23to1.84)

Vanuatu	0(0-0)	0.051(0.012-0.106)	0(0-0)	0.037(0.01-0.073)	-1.08(-1.15to-1.01)
Venezuela (Bolivarian Republic of)	4(1-7)	0.042(0.015-0.077)	10(3-19)	0.035(0.011-0.066)	-0.86(-1.29to-0.43)
Viet Nam	31(10-58)	0.082(0.026-0.152)	76(26-147)	0.082(0.028-0.156)	-0.43(-0.61to-0.26)
Yemen	13(3-29)	0.266(0.066-0.603)	38(11-78)	0.287(0.083-0.584)	0.36(0.26to0.46)
Zambia	0(0-0)	0.007(0.002-0.014)	0(0-1)	0.007(0.002-0.013)	-0.22(-0.37to-0.08)
Zimbabwe	3(1-8)	0.096(0.031-0.212)	5(2-10)	0.075(0.026-0.166)	-0.7(-0.94to-0.45)
**DALYs**					
Afghanistan	210(43-568)	3.003(0.622-8.012)	338(91-795)	3.456(0.891-8.133)	0.64(0.36to0.92)
Albania	121(41-244)	5.793(1.921-11.688)	218(68-441)	4.862(1.522-9.887)	-0.26(-0.39to-0.14)
Algeria	314(101-712)	2.656(0.834-5.593)	745(219-1831)	2.183(0.631-5.019)	-0.55(-0.62to-0.47)
American Samoa	0(0-1)	1.789(0.464-3.627)	0(0-1)	0.662(0.222-1.399)	-3.99(-4.42to-3.55)
Andorra	9(3-18)	15.45(5.118-30.787)	12(4-26)	7.892(2.388-17.079)	-1.91(-2.11to-1.72)
Angola	25(7-55)	0.617(0.158-1.389)	74(21-164)	0.562(0.159-1.279)	-0.21(-0.46to0.03)
Antigua and Barbuda	1(0-2)	1.701(0.521-3.209)	2(1-4)	1.916(0.604-3.637)	0.5(0.27to0.73)
Argentina	1400(502-2355)	4.249(1.53-7.167)	2053(746-3533)	3.708(1.363-6.339)	-0.17(-0.55to0.21)
Armenia	108(45-186)	3.467(1.44-5.998)	218(94-337)	4.991(2.151-7.717)	1.67(1to2.34)
Australia	1380(483-2352)	6.898(2.419-11.742)	2442(755-4765)	5.303(1.683-10.201)	-1.11(-1.48to-0.73)
Austria	651(239-1125)	5.718(2.121-9.863)	1241(427-2156)	7.04(2.486-12.109)	1.24(0.88to1.61)
Azerbaijan	179(66-358)	3.199(1.184-6.403)	368(121-825)	3.117(1.036-6.852)	0.17(-0.02to0.35)
Bahamas	2(1-4)	1.13(0.333-2.428)	5(2-10)	1.322(0.387-2.557)	1.06(0.83to1.3)
Bahrain	17(5-33)	9.817(2.743-19.356)	55(16-128)	6.092(1.814-13.058)	-2.02(-2.21to-1.82)
Bangladesh	559(160-1099)	1.179(0.343-2.35)	1092(340-2270)	0.793(0.251-1.616)	-1.38(-1.47to-1.28)
Barbados	3(1-6)	1.169(0.395-2.084)	6(2-12)	1.204(0.392-2.389)	0.23(-0.03to0.5)
Belarus	372(137-690)	2.819(1.024-5.202)	721(288-1249)	4.59(1.847-7.955)	1.49(1.08to1.89)
Belgium	1593(614-2548)	10.425(3.995-16.713)	2217(760-3914)	9.672(3.379-16.98)	-0.09(-0.41to0.23)

Belize	0(0-0)	0.15(0.051-0.274)	1(0-2)	0.3(0.108-0.52)	2.28(1.58to2.99)
Benin	1(0-2)	0.056(0.019-0.119)	2(1-5)	0.043(0.012-0.084)	-0.99(-1.23to-0.75)
Bermuda	2(1-4)	2.813(0.868-5.707)	3(1-5)	2.032(0.661-3.867)	-0.6(-0.87to-0.33)
Bhutan	2(0-5)	0.853(0.178-2.198)	5(1-13)	0.806(0.193-2.109)	-0.21(-0.23to-0.18)
Bolivia (Plurinational State of)	33(10-71)	0.982(0.309-2.127)	83(26-167)	0.884(0.271-1.795)	0.02(-0.11to0.14)
Bosnia and Herzegovina	238(87-440)	5.544(2.019-10.19)	397(139-743)	6.308(2.213-11.907)	0.89(0.6to1.17)
Botswana	9(3-19)	1.513(0.439-3.263)	19(6-38)	1.24(0.37-2.506)	-0.83(-1to-0.66)
Brazil	4930(1863-8236)	5.268(1.987-8.929)	8156(2629-14822)	3.245(1.04-5.9)	-1.71(-1.83to-1.6)
Brunei Darussalam	12(4-21)	10.526(3.99-19.134)	21(7-39)	5.532(1.709-10.286)	-1.64(-1.84to-1.43)
Bulgaria	733(286-1214)	5.823(2.273-9.655)	1139(418-1931)	8.831(3.285-14.802)	1.98(1.64to2.31)
Burkina Faso	2(1-4)	0.04(0.013-0.08)	5(2-10)	0.048(0.014-0.093)	0.69(0.62to0.75)
Burundi	6(1-13)	0.231(0.057-0.508)	8(2-19)	0.14(0.035-0.318)	-1.56(-1.93to-1.18)
Cabo Verde	0(0-1)	0.204(0.069-0.525)	1(0-2)	0.219(0.068-0.459)	-0.36(-0.69to-0.02)
Cambodia	239(67-493)	5.41(1.544-11.08)	603(202-1094)	4.876(1.71-8.825)	-0.55(-0.62to-0.47)
Cameroon	4(1-8)	0.073(0.024-0.154)	11(3-21)	0.073(0.02-0.146)	0.14(-0.06to0.34)
Canada	4001(1438-6529)	12.311(4.414-20.166)	6078(1921-10937)	8.192(2.636-14.592)	-0.88(-1.04to-0.72)
Central African Republic	5(1-10)	0.351(0.099-0.823)	7(2-14)	0.248(0.074-0.509)	-1.22(-1.39to-1.04)
Chad	2(1-4)	0.057(0.017-0.126)	5(1-10)	0.075(0.021-0.161)	0.86(0.61to1.12)
Chile	222(87-383)	2.048(0.791-3.536)	376(140-650)	1.526(0.57-2.635)	-0.67(-0.9to-0.44)
China	20282(6820-37075)	2.294(0.785-4.194)	46222(16687-85952)	2.136(0.758-3.958)	-0.3(-0.39to-0.21)
Colombia	202(79-351)	1.029(0.402-1.811)	365(129-668)	0.667(0.235-1.224)	-1.94(-2.12to-1.76)
Comoros	1(0-1)	0.28(0.073-0.601)	1(0-2)	0.214(0.063-0.46)	-1.18(-1.33to-1.02)
Congo	5(1-9)	0.408(0.129-0.838)	11(3-21)	0.361(0.111-0.701)	-0.31(-0.46to-0.15)
Cook Islands	0(0-0)	1.037(0.249-2.281)	0(0-0)	0.714(0.188-1.508)	-1.22(-1.27to-1.17)
Costa Rica	49(18-88)	2.791(0.985-5.019)	125(43-219)	2.291(0.782-4.023)	-0.75(-0.98to-0.51)
Croatia	470(167-764)	7.46(2.694-12.173)	754(277-1255)	8.493(3.138-14.063)	1.05(0.84to1.26)

Cuba	395(145-684)	3.855(1.417-6.673)	606(226-1031)	3.188(1.192-5.433)	-0.6(-0.76to-0.44)
Cyprus	108(38-202)	13.197(4.806-24.878)	210(68-370)	9.833(3.187-17.267)	-0.46(-0.62to-0.31)
Czechia	1166(422-1976)	8.384(3.039-14.191)	1308(471-2273)	5.965(2.195-10.369)	-0.34(-0.67to-0.01)
Côte d'Ivoire	5(1-11)	0.129(0.036-0.275)	12(4-24)	0.106(0.03-0.221)	-1.03(-1.35to-0.72)
Democratic People's Republic of Korea	378(132-787)	2.134(0.747-4.369)	692(228-1543)	2.031(0.678-4.447)	-0.19(-0.3to-0.08)
Democratic Republic of the Congo	35(10-69)	0.189(0.056-0.378)	73(22-156)	0.156(0.047-0.332)	-0.54(-0.8to-0.28)
Denmark	1407(558-2237)	17.471(6.904-27.871)	1262(440-2194)	10.124(3.552-17.68)	-1.73(-2to-1.46)
Djibouti	1(0-2)	0.422(0.102-1.003)	3(1-6)	0.373(0.104-0.793)	-0.23(-0.37to-0.08)
Dominica	0(0-1)	0.705(0.229-1.358)	0(0-1)	0.524(0.177-1.044)	-0.98(-1.06to-0.89)
Dominican Republic	73(24-179)	1.953(0.647-4.922)	184(59-378)	1.847(0.593-3.813)	0.05(-0.26to0.36)
Ecuador	49(17-87)	0.902(0.311-1.617)	128(47-234)	0.774(0.284-1.413)	0.31(-0.18to0.81)
Egypt	1278(371-3337)	4.413(1.251-12.562)	5042(1671-10705)	7.58(2.38-16.684)	2.43(2.09to2.77)
El Salvador	20(6-41)	0.631(0.196-1.331)	53(15-102)	0.87(0.251-1.689)	1.17(1.03to1.31)
Equatorial Guinea	1(0-1)	0.272(0.084-0.569)	2(1-4)	0.263(0.075-0.553)	-0.03(-0.29to0.24)
Eritrea	3(1-6)	0.184(0.046-0.432)	6(2-14)	0.166(0.04-0.382)	-0.6(-0.73to-0.47)
Estonia	46(16-82)	2.272(0.801-4.089)	109(38-186)	4.468(1.609-7.659)	2.2(1.21to3.21)
Eswatini	2(1-4)	0.569(0.193-1.139)	3(1-7)	0.463(0.15-1.011)	-0.5(-0.87to-0.13)
Ethiopia	57(14-120)	0.238(0.06-0.501)	84(20-199)	0.163(0.039-0.393)	-1.04(-1.31to-0.77)
Fiji	18(6-32)	4.712(1.503-8.644)	21(6-43)	2.56(0.745-5.235)	-1.99(-2.21to-1.77)
Finland	332(113-610)	4.683(1.61-8.629)	357(111-679)	2.886(0.926-5.358)	-1.45(-1.6to-1.3)
France	5616(2015-9647)	6.947(2.51-11.872)	7576(2541-13669)	5.384(1.868-9.561)	-0.33(-0.59to-0.08)
Gabon	2(1-4)	0.308(0.09-0.64)	4(1-7)	0.302(0.089-0.595)	-0.12(-0.2to-0.03)
Gambia	0(0-1)	0.071(0.022-0.156)	1(0-1)	0.05(0.016-0.107)	-1.47(-1.65to-1.29)
Georgia	204(76-354)	3.171(1.189-5.489)	320(131-521)	5.783(2.35-9.442)	3.59(2.62to4.56)

Germany	10405(3774-17747)	8.477(3.078-14.529)	15758(5460-27383)	8.262(2.906-14.249)	0.16(0.03to0.3)
Ghana	7(2-14)	0.114(0.032-0.219)	14(5-30)	0.074(0.025-0.162)	-1.66(-2to-1.32)
Greece	1737(699-2711)	11.302(4.554-17.646)	3299(1282-5351)	14.57(5.656-23.424)	0.85(0.64to1.06)
Greenland	3(1-6)	9.257(3.257-17.661)	3(1-6)	4.145(1.158-8.647)	-2.53(-2.66to-2.39)
Grenada	0(0-1)	0.447(0.138-0.858)	1(0-1)	0.495(0.175-0.874)	-0.07(-0.44to0.3)
Guam	1(0-2)	1.361(0.472-2.857)	3(1-6)	1.764(0.623-3.249)	1.27(0.76to1.78)
Guatemala	11(4-20)	0.281(0.094-0.522)	43(16-74)	0.356(0.131-0.617)	0.46(0.13to0.78)
Guinea	1(0-2)	0.024(0.008-0.052)	2(0-3)	0.025(0.008-0.055)	0.25(0.16to0.33)
Guinea-Bissau	0(0-0)	0.045(0.014-0.113)	1(0-1)	0.078(0.024-0.165)	2.36(2.14to2.57)
Guyana	1(0-1)	0.18(0.06-0.33)	1(0-3)	0.207(0.07-0.385)	0.71(0.45to0.97)
Haiti	22(7-46)	0.573(0.177-1.225)	33(10-67)	0.376(0.116-0.766)	-1.37(-1.48to-1.25)
Honduras	29(9-59)	1.318(0.411-2.727)	92(29-200)	1.397(0.43-3.075)	0.29(0.2to0.38)
Hungary	1282(495-2112)	8.769(3.398-14.383)	1417(504-2511)	7.571(2.728-13.458)	-0.3(-0.54to-0.05)
Iceland	29(11-48)	10.452(3.762-17.253)	48(15-84)	8.257(2.694-14.473)	-0.87(-0.99to-0.75)
India	3369(1080-6580)	0.712(0.225-1.389)	6996(2339-12517)	0.591(0.196-1.059)	-0.73(-0.79to-0.68)
Indonesia	1735(542-3281)	1.67(0.525-3.21)	4934(1669-8855)	1.984(0.682-3.563)	0.43(0.23to0.64)
Iran (Islamic Republic of)	1405(409-2581)	4.938(1.43-9.222)	3021(858-5476)	3.78(1.048-6.922)	-0.58(-0.69to-0.48)
Iraq	533(159-1365)	6.592(1.952-17.079)	1453(444-3384)	5.76(1.715-13.891)	-0.55(-0.66to-0.44)
Ireland	401(154-646)	9.408(3.6-15.224)	410(141-729)	5.029(1.75-8.93)	-1.94(-2.33to-1.55)
Israel	439(165-787)	9.046(3.442-16.197)	902(311-1593)	7.273(2.476-12.776)	-0.44(-0.71to-0.18)
Italy	5866(2109-9780)	6.686(2.411-11.147)	10124(3458-17614)	7.149(2.508-12.366)	0.41(0.13to0.69)
Jamaica	10(3-18)	0.56(0.196-1.02)	33(11-66)	1.099(0.347-2.153)	2.53(1.87to3.19)
Japan	12796(5135-20235)	7.426(2.972-11.758)	15122(5339-26150)	4.391(1.62-7.469)	-1.51(-1.79to-1.24)
Jordan	311(118-523)	21.721(8.34-36.747)	1063(390-1890)	13.6(4.941-24.307)	-1.54(-1.7to-1.38)
Kazakhstan	338(108-639)	2.445(0.783-4.6)	291(110-529)	1.486(0.558-2.712)	-1.22(-1.72to-0.72)
Kenya	15(4-35)	0.176(0.049-0.403)	39(13-75)	0.153(0.051-0.288)	-0.64(-0.75to-0.53)

Kiribati	1(0-2)	1.756(0.331-3.772)	2(0-4)	2.206(0.431-4.868)	0.73(0.6to0.86)
Kuwait	35(13-62)	5.175(1.849-8.947)	113(40-192)	3.683(1.273-6.377)	-0.8(-1.49to-0.1)
Kyrgyzstan	58(22-99)	1.798(0.67-3.117)	140(57-235)	2.487(1.001-4.135)	1.71(1.33to2.08)
Lao People's Democratic Republic	88(25-195)	4.197(1.188-9.213)	145(48-271)	3.175(1.074-5.806)	-0.91(-0.95to-0.86)
Latvia	158(64-272)	4.509(1.815-7.733)	121(46-207)	3.511(1.346-5.971)	-0.76(-1.1to-0.42)
Lebanon	244(88-514)	11.061(4.073-23.108)	626(221-1089)	10.474(3.715-18.092)	0.2(-0.01to0.42)
Lesotho	17(5-38)	1.991(0.633-4.567)	33(10-68)	2.924(0.917-6.031)	1.83(1.66to2)
Liberia	1(0-1)	0.052(0.017-0.107)	2(0-3)	0.056(0.016-0.109)	0.47(0.34to0.61)
Libya	138(43-320)	7.032(2.155-16.547)	325(103-708)	5.979(1.866-12.741)	-0.23(-0.37to-0.1)
Lithuania	67(24-123)	1.511(0.543-2.741)	191(71-332)	3.678(1.394-6.275)	3.49(2.76to4.22)
Luxembourg	64(22-114)	11.769(4.172-21.195)	109(31-200)	10.37(2.997-19.032)	-0.35(-0.59to-0.11)
Madagascar	9(3-19)	0.165(0.043-0.343)	12(3-26)	0.085(0.022-0.18)	-2.16(-2.39to-1.92)
Malawi	3(1-6)	0.071(0.017-0.159)	6(2-12)	0.069(0.019-0.139)	-0.3(-0.49to-0.11)
Malaysia	255(79-462)	2.662(0.826-4.927)	552(184-1030)	1.931(0.643-3.633)	-1.18(-1.46to-0.91)
Maldives	6(2-12)	7.207(2.222-14.493)	12(4-23)	3.965(1.454-7.058)	-2.19(-2.3to-2.08)
Mali	1(0-2)	0.03(0.009-0.059)	3(1-7)	0.036(0.013-0.074)	0.84(0.77to0.91)
Malta	38(14-64)	8.625(3.14-14.841)	74(26-133)	8.064(2.824-14.196)	0(-0.19to0.19)
Marshall Islands	0(0-1)	2.061(0.51-4.498)	1(0-2)	2.299(0.563-5.164)	0.57(0.45to0.69)
Mauritania	1(0-2)	0.081(0.027-0.179)	2(1-4)	0.082(0.025-0.159)	0.08(-0.02to0.18)
Mauritius	2(1-3)	0.24(0.093-0.412)	13(5-21)	0.708(0.274-1.181)	10.9(5.2to16.9)
Mexico	537(201-886)	1.152(0.432-1.924)	798(297-1346)	0.613(0.227-1.035)	-2.41(-2.55to-2.27)
Micronesia (Federated States of)	1(0-2)	2.253(0.686-4.508)	2(1-4)	2.216(0.661-4.441)	0.02(-0.02to0.06)
Monaco	10(3-19)	15.522(5.096-28.841)	14(4-30)	15.553(3.908-32.577)	0.09(-0.13to0.32)
Mongolia	14(5-27)	1.252(0.462-2.365)	41(13-71)	1.437(0.475-2.56)	0.63(0.54to0.71)

Montenegro	50(17-94)	7.833(2.697-14.522)	73(27-132)	7.185(2.617-12.996)	-0.16(-0.39to0.07)
Morocco	142(42-358)	0.97(0.279-2.466)	273(86-648)	0.769(0.235-1.829)	-0.71(-0.86to-0.55)
Mozambique	14(4-31)	0.219(0.058-0.473)	27(8-58)	0.206(0.061-0.429)	0.18(0.07to0.3)
Myanmar	807(263-1746)	3.484(1.154-7.369)	696(265-1241)	1.445(0.548-2.568)	-3.15(-3.31to-3)
Namibia	4(1-7)	0.552(0.2-1.043)	6(2-12)	0.428(0.152-0.793)	-1.08(-1.36to-0.8)
Nauru	0(0-0)	2.623(0.715-5.737)	0(0-0)	2.08(0.545-4.413)	-0.86(-0.95to-0.78)
Nepal	69(22-137)	0.722(0.232-1.39)	116(38-216)	0.495(0.167-0.92)	-1.54(-1.67to-1.4)
Netherlands	2459(931-4015)	12.474(4.766-20.366)	2689(885-4826)	7.28(2.41-12.919)	-1.55(-1.71to-1.38)
New Zealand	324(111-561)	8.083(2.781-13.927)	319(105-587)	3.74(1.247-6.789)	-2.54(-2.83to-2.26)
Nicaragua	11(4-21)	0.61(0.202-1.241)	30(11-57)	0.576(0.202-1.077)	0.11(-0.05to0.26)
Niger	1(0-2)	0.033(0.009-0.071)	3(1-7)	0.036(0.009-0.08)	0.32(0.15to0.49)
Nigeria	23(7-46)	0.051(0.016-0.102)	37(12-72)	0.039(0.012-0.077)	-0.95(-1.02to-0.88)
Niue	0(0-0)	2.23(0.56-4.944)	0(0-0)	1.972(0.477-4.505)	-0.53(-0.59to-0.47)
North Macedonia	180(66-342)	9.319(3.389-17.827)	270(85-498)	7.81(2.425-14.428)	-0.59(-0.82to-0.35)
Northern Mariana Islands	1(0-1)	3.064(0.923-5.82)	0(0-1)	0.841(0.252-2.34)	-4.78(-5.16to-4.41)
Norway	486(181-819)	7.275(2.712-12.083)	356(116-645)	3.497(1.171-6.282)	-2.56(-2.74to-2.38)
Oman	21(5-47)	2.887(0.676-6.325)	36(12-72)	1.591(0.474-3.322)	-1.43(-1.62to-1.23)
Pakistan	916(260-1786)	1.655(0.473-3.234)	1200(378-2189)	1.016(0.321-1.864)	-1.9(-2.11to-1.68)
Palau	0(0-0)	0.31(0.051-0.71)	0(0-0)	0.243(0.043-0.613)	-0.84(-0.89to-0.8)
Palestine	24(7-50)	2.712(0.801-5.587)	58(17-116)	2.145(0.632-4.179)	-1.1(-1.28to-0.91)
Panama	18(6-32)	1.183(0.403-2.154)	48(16-90)	1.092(0.37-2.056)	-0.5(-0.67to-0.33)
Papua New Guinea	22(4-49)	0.954(0.182-2.111)	61(14-137)	0.868(0.197-1.941)	-0.44(-0.57to-0.32)
Paraguay	61(22-118)	2.763(0.994-5.312)	127(39-248)	2.205(0.681-4.314)	-0.76(-0.92to-0.59)
Peru	58(18-113)	0.451(0.136-0.886)	173(50-346)	0.5(0.144-0.998)	0.28(0.1to0.46)
Philippines	1394(484-2458)	4.629(1.616-8.184)	2464(910-4463)	2.902(1.061-5.219)	-1.83(-2.07to-1.59)
Poland	3194(1225-5331)	7.148(2.748-11.926)	4079(1459-6945)	5.592(2.018-9.509)	-0.22(-0.62to0.19)

Portugal	609(204-1075)	4.329(1.457-7.635)	880(287-1559)	3.802(1.304-6.646)	-0.33(-0.61to-0.05)
Puerto Rico	115(35-244)	3.133(0.96-6.653)	200(56-438)	2.936(0.844-6.365)	-0.43(-0.65to-0.21)
Qatar	8(2-16)	5.772(1.524-12.339)	26(8-56)	2.21(0.684-4.191)	-3.18(-3.54to-2.83)
Republic of Korea	2583(989-4657)	7.896(3.018-14.661)	4491(1115-8420)	4.767(1.187-8.93)	-1.64(-1.74to-1.53)
Republic of Moldova	252(91-468)	5.372(1.939-9.994)	317(116-514)	5.362(1.949-8.72)	0.54(0.04to1.04)
Romania	698(260-1179)	2.452(0.911-4.144)	1332(457-2257)	3.808(1.332-6.414)	1.63(1.44to1.81)
Russian Federation	4174(1679-7029)	2.253(0.901-3.784)	7027(2804-11320)	2.98(1.192-4.8)	1.15(0.83to1.47)
Rwanda	11(3-24)	0.367(0.11-0.779)	24(7-51)	0.363(0.112-0.757)	-0.45(-0.61to-0.29)
Saint Kitts and Nevis	0(0-0)	0.248(0.075-0.454)	0(0-0)	0.239(0.07-0.482)	0.25(0to0.51)
Saint Lucia	1(0-2)	1.031(0.362-1.911)	2(1-4)	0.8(0.265-1.484)	-0.85(-1.13to-0.57)
Saint Vincent and the Grenadines	0(0-0)	0.321(0.109-0.593)	1(0-1)	0.523(0.178-0.941)	1.78(1.47to2.08)
Samoa	5(1-10)	5.626(1.595-11.297)	8(2-15)	5.377(1.582-10.668)	-0.33(-0.41to-0.25)
San Marino	3(1-5)	7.379(2.503-14.623)	2(1-5)	3.45(0.932-7.57)	-1.55(-1.85to-1.24)
Sao Tome and Principe	0(0-0)	0.021(0.007-0.041)	0(0-0)	0.026(0.008-0.055)	0.51(0.13to0.9)
Saudi Arabia	106(29-268)	1.579(0.426-4.182)	496(137-1057)	2.06(0.544-4.077)	1.17(0.66to1.69)
Senegal	3(1-7)	0.099(0.03-0.206)	9(2-18)	0.106(0.027-0.221)	0.21(0.11to0.32)
Serbia	660(226-1167)	5.681(1.905-10.009)	1004(335-1837)	6.243(2.095-11.356)	0.63(0.37to0.88)
Seychelles	1(0-3)	2.116(0.645-4.435)	2(1-4)	1.623(0.445-3.123)	-0.93(-1.11to-0.74)
Sierra Leone	2(1-3)	0.072(0.024-0.143)	3(1-6)	0.069(0.02-0.127)	0.2(0.04to0.35)
Singapore	126(44-225)	5.5(1.903-9.894)	175(61-321)	2.012(0.699-3.671)	-2.72(-3.13to-2.31)
Slovakia	358(130-696)	5.966(2.168-11.589)	353(101-692)	3.701(1.068-7.25)	-1.35(-1.42to-1.27)
Slovenia	137(50-236)	5.569(2.065-9.597)	231(78-421)	5.468(1.908-9.894)	0.66(0.34to0.98)
Solomon Islands	4(1-7)	2.416(0.524-4.854)	10(3-19)	2.52(0.633-4.858)	0.32(0.17to0.47)
Somalia	6(1-14)	0.188(0.044-0.451)	13(3-28)	0.155(0.039-0.362)	-0.63(-0.69to-0.57)
South Africa	472(160-862)	2.148(0.708-4.049)	518(185-939)	1.046(0.368-1.882)	-2.4(-2.49to-2.3)

South Sudan	6(2-14)	0.231(0.058-0.516)	8(2-17)	0.182(0.048-0.386)	-0.89(-1.03to-0.74)
Spain	3464(1388-5508)	6.574(2.63-10.461)	5589(1960-9590)	5.992(2.15-10.216)	-0.26(-0.59to0.07)
Sri Lanka	39(11-112)	0.354(0.097-1.014)	108(25-226)	0.402(0.096-0.842)	1.67(0.96to2.37)
Sudan	511(138-1098)	5.249(1.388-11.142)	1053(261-2459)	5.085(1.241-12.39)	-0.14(-0.2to-0.09)
Suriname	4(2-8)	1.599(0.576-3.227)	9(3-17)	1.324(0.396-2.583)	-0.31(-0.59to-0.04)
Sweden	1084(405-1910)	7.266(2.715-12.71)	1451(468-2607)	6.502(2.187-11.415)	-0.56(-0.72to-0.39)
Switzerland	997(364-1691)	9.978(3.663-16.878)	1132(392-1962)	6.17(2.135-10.611)	-0.95(-1.25to-0.66)
Syrian Arab Republic	451(140-950)	8.248(2.57-17.621)	751(232-1739)	5.54(1.687-12.91)	-1.54(-1.67to-1.42)
Taiwan	882(347-1456)	5.273(2.096-8.632)	2222(815-3616)	5.26(1.936-8.532)	-0.13(-0.52to0.26)
Tajikistan	108(33-227)	3.616(1.091-7.585)	116(30-252)	1.61(0.445-3.38)	-2.59(-2.78to-2.39)
Thailand	1512(509-2788)	4.325(1.446-8.026)	3454(765-6994)	3.268(0.73-6.588)	-1.02(-1.14to-0.9)
Timor-Leste	5(1-12)	1.689(0.426-3.915)	12(3-26)	1.462(0.401-3.082)	-0.46(-0.55to-0.36)
Togo	3(1-6)	0.23(0.062-0.561)	8(2-17)	0.216(0.052-0.452)	-0.24(-0.3to-0.17)
Tokelau	0(0-0)	2.198(0.513-5.001)	0(0-0)	1.761(0.429-3.677)	-0.76(-0.8to-0.72)
Tonga	2(1-4)	3.89(1.116-8.163)	3(1-6)	3.621(1.046-7.505)	-0.29(-0.37to-0.22)
Trinidad and Tobago	16(6-28)	1.785(0.637-3.187)	30(10-58)	1.587(0.521-3.008)	-0.28(-0.46to-0.1)
Tunisia	219(70-547)	4.255(1.344-10.675)	464(151-1124)	3.403(1.098-8.279)	-0.85(-0.93to-0.78)
Turkey	5516(1969-9728)	14.911(5.422-25.803)	8220(2940-14599)	8.517(3.039-15.157)	-1.96(-2.13to-1.8)
Turkmenistan	62(25-109)	2.839(1.111-5.02)	86(28-161)	1.838(0.589-3.437)	-1.51(-1.73to-1.3)
Tuvalu	0(0-0)	2.062(0.479-4.457)	0(0-0)	1.981(0.601-3.905)	-0.05(-0.08to-0.02)
Uganda	7(2-15)	0.102(0.028-0.209)	18(5-40)	0.1(0.03-0.219)	-0.62(-0.97to-0.27)
Ukraine	2885(1129-4992)	4.06(1.583-7.005)	1563(536-2818)	2.123(0.739-3.81)	-2.76(-3.06to-2.46)
United Arab Emirates	31(9-63)	5.999(1.436-12.977)	136(39-285)	2.935(0.855-6.06)	-1.36(-1.75to-0.97)
United Kingdom	10437(3898-16768)	11.638(4.376-18.786)	11901(3912-21112)	8.846(2.967-15.487)	-1(-1.21to-0.8)
United Republic of Tanzania	39(10-81)	0.318(0.083-0.671)	73(19-155)	0.237(0.063-0.499)	-1.24(-1.35to-1.13)
United States Virgin Islands	1(0-3)	1.53(0.406-3.369)	1(0-3)	0.761(0.194-1.758)	-2.16(-2.37to-1.94)

United States of America	51335(19047-83814)	16.654(6.197-27.119)	73196(23412-128999)	12.282(3.993-21.499)	-1.04(-1.31to-0.76)
Uruguay	163(60-276)	4.275(1.575-7.209)	247(89-427)	4.914(1.807-8.359)	0.27(0.08to0.46)
Uzbekistan	80(27-159)	0.638(0.215-1.273)	255(95-455)	0.847(0.309-1.509)	0.9(0.57to1.22)
Vanuatu	1(0-2)	1.238(0.292-2.423)	2(1-4)	0.99(0.269-1.933)	-0.79(-0.87to-0.72)
Venezuela (Bolivarian Republic of)	118(43-205)	1.085(0.394-1.919)	256(87-473)	0.845(0.288-1.561)	-1.12(-1.48to-0.76)
Viet Nam	716(225-1392)	1.781(0.561-3.396)	1868(644-3671)	1.842(0.64-3.541)	-0.18(-0.3to-0.06)
Yemen	362(92-806)	6.785(1.708-15.207)	1030(282-2129)	6.858(1.911-14.203)	0.12(0.02to0.22)
Zambia	6(2-11)	0.179(0.055-0.352)	16(4-32)	0.184(0.051-0.364)	0.07(-0.07to0.21)
Zimbabwe	83(25-185)	2.052(0.645-4.587)	126(44-264)	1.708(0.601-3.662)	-0.57(-0.84to-0.3)

DALYs: disability-adjusted life years.

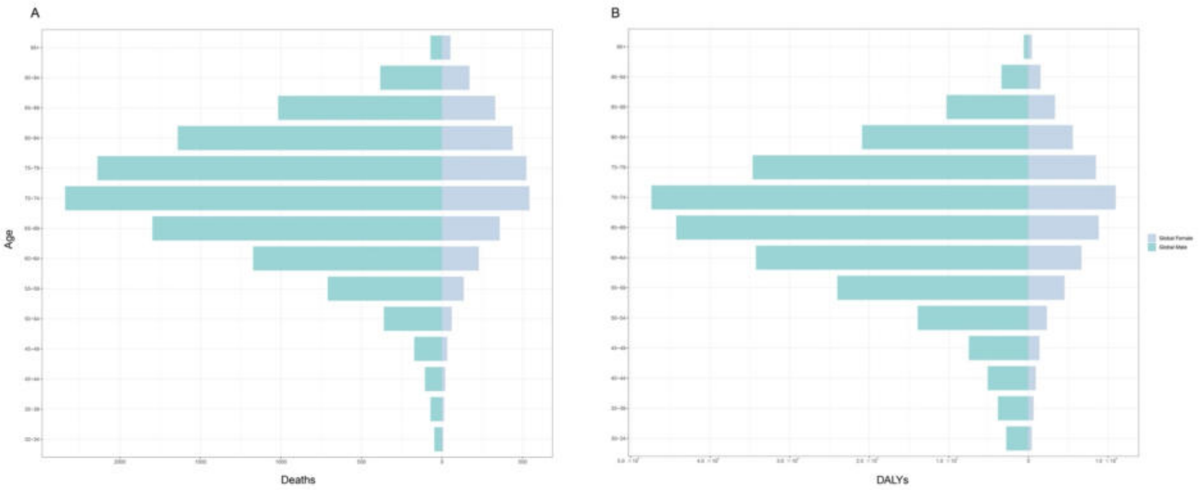

Supplementary Figure 1: The number of global deaths and DALYs of AML attributable to tobacco by age and sex in 2021, based on Global Burden of Disease 2021 data. (A) Deaths. (B) DALYs. AML: acute myeloid leukemia; DALYs: disability-adjusted life years.

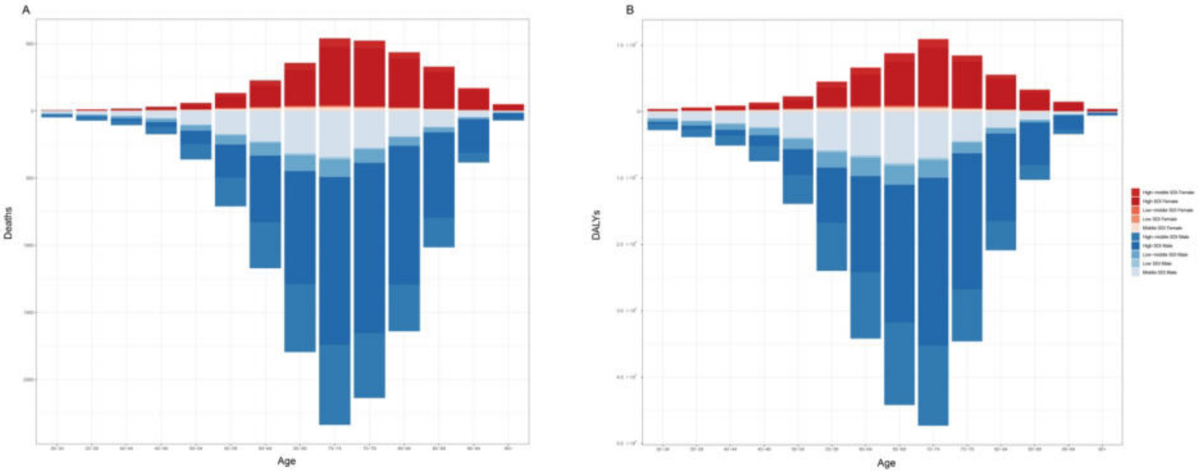

Supplementary Figure 2: The number of global deaths and DALYs of AML attributable to tobacco by age and sex across SDI regions in 2021, based on Global Burden of Disease 2021 data. (A) Deaths. (B) DALYs. AML: acute myeloid leukemia; SDI: socio-demographic index; DALYs: disability-adjusted life years.

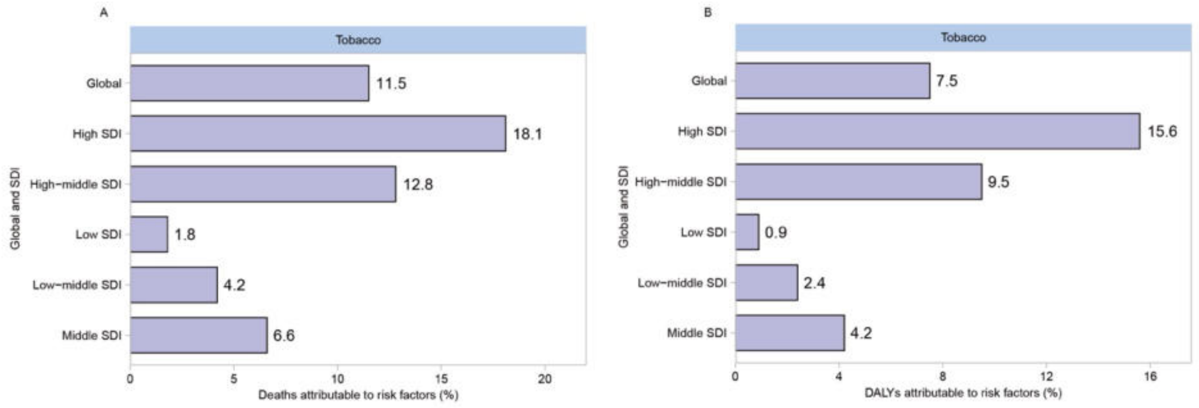

Supplementary Figure 3: Proportion of AML attributable to tobacco deaths and DALYs in 2021 in global and SDI regions, based on Global Burden of Disease 2021 data. (A) Deaths. (B) DALYs. AML: acute myeloid leukemia; DALYs: disability-adjusted life years.

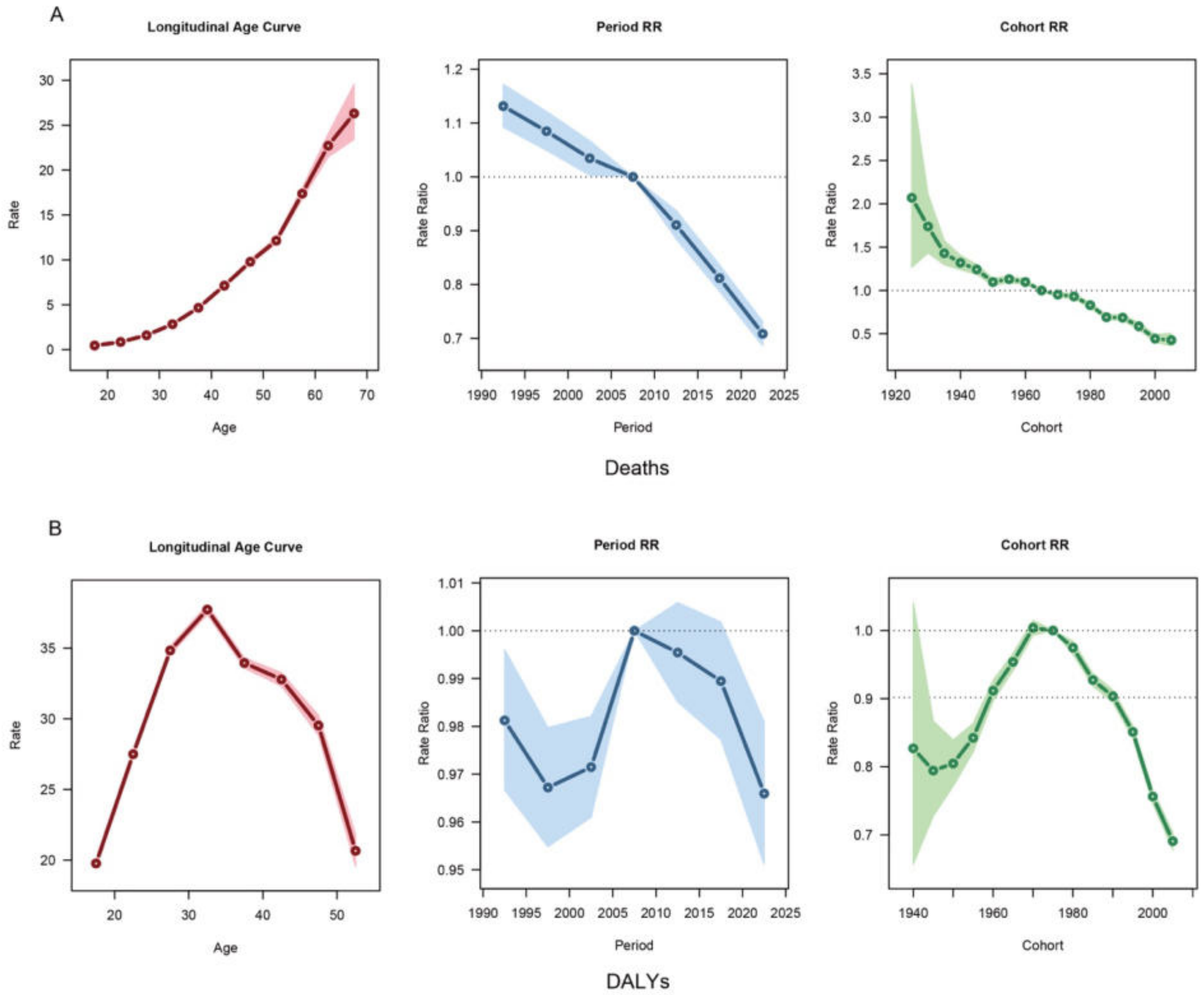

Supplementary Figure 4: Age, period, and cohort effects on AML attributable to tobacco global deaths and DALYs during 1990-2021, based on Global Burden of Disease 2021 data. (A) Deaths. (B) DALYs. AML: acute myeloid leukemia; DALYs: disability-adjusted life years

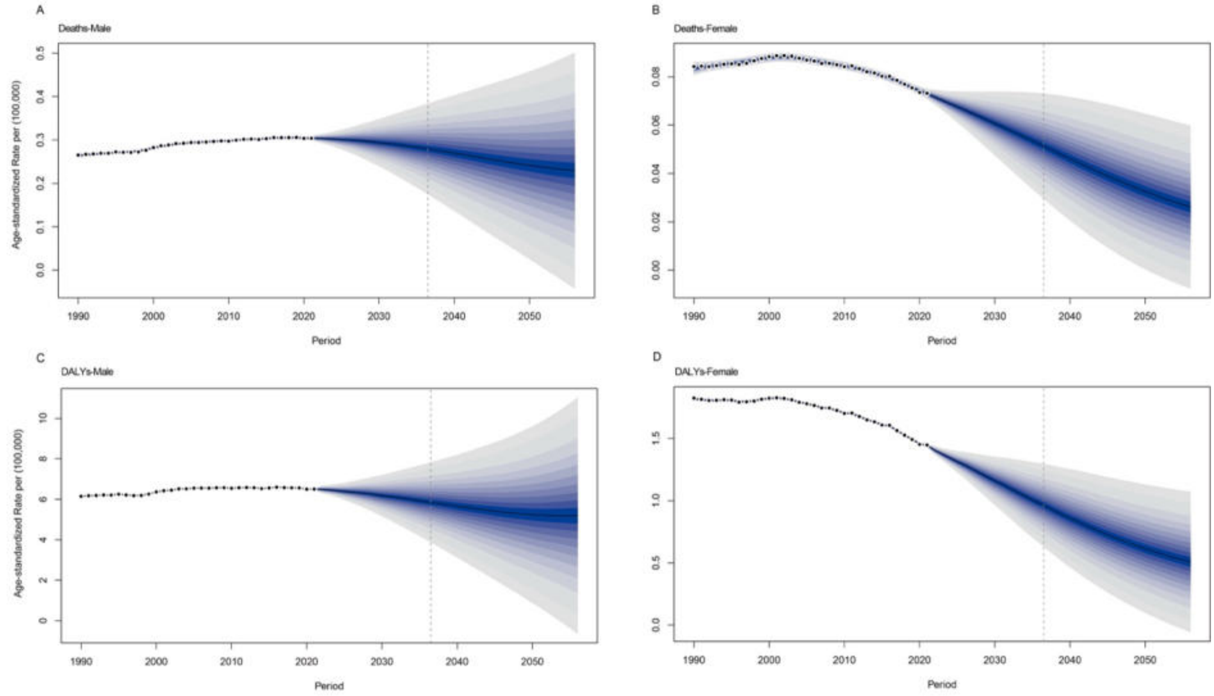

Supplementary Figure 5: Future projections of the global deaths and DALYs of AML attributable to tobacco by sex, based on Global Burden of Disease 2021 data. (A) Male deaths. (B) Female deaths. (C) Male DALYs. (D) Female DALYs. AML: acute myeloid leukemia; DALYs: disability-adjusted life years.
